# Sustained-Release Oral Delivery of NSAIDs and Acetaminophen: Advances and Recent Formulation Strategies—A Systematic Review

**DOI:** 10.3390/pharmaceutics17101264

**Published:** 2025-09-26

**Authors:** Paulina Drapińska, Katarzyna Skulmowska-Polok, Joanna Chałupka, Adam Sikora

**Affiliations:** 1 Department of Pharmaceutical Technology, Faculty of Pharmacy, Medical Biotechnology and Laboratory Medicine, Pomeranian Medical University in Szczecin, 71-251 Szczecin, Poland; paulina.drapinska@pum.edu.pl (P.D.); katarzyna.skulmowska.polok@pum.edu.pl (K.S.-P.); joanna.chalupka@pum.edu.pl (J.C.); 2 Department of Medicinal Chemistry, Faculty of Pharmacy, Collegium Medicum in Bydgoszcz, Nicolaus Copernicus University in Toruń, Dr. A. Jurasza 2, 85-089 Bydgoszcz, Poland

**Keywords:** modified-release dosage forms, non-steroidal anti-inflammatory drugs, mucoadhesive polymers, hot-melt extrusion, 3D printing, nanocarriers

## Abstract

**Background**: Sustained-release (SR) formulations of non-steroidal anti-inflammatory drugs (NSAIDs) aim to prolong therapeutic activity, reduce dosing frequency, and improve patient adherence. However, currently marketed SR NSAIDs exhibit persistent limitations, including incomplete control over release kinetics, high interpatient variability in bioavailability, limited reduction in gastrointestinal adverse effects, and insufficient dose flexibility for individualized therapy. In many cases, conventional excipients and release mechanisms remain predominant, leaving drug-specific physicochemical and pharmacokinetic constraints only partially addressed. These gaps highlight the need for a comprehensive synthesis of recent technological advances to guide the development of more effective, patient-centered delivery systems. **Methods**: A narrative literature review was conducted using Web of Science and PubMed databases to identify original research articles and comprehensive technological studies on oral SR formulations of NSAIDs and paracetamol published between January 2020 and March 2025. Inclusion criteria focused on preclinical and technological research addressing formulation design, excipient innovations, and manufacturing approaches. **Results**: Sixty-four studies met the inclusion criteria, encompassing polymeric matrices (31%), lipid-based carriers (18%), microspheres/hydrogel beads/interpenetrating polymer networks (30%), nanostructured systems (11%), and hybrid platforms (10%). The most common strategies involved pH-dependent release, mucoadhesive systems, and floating drug delivery, aiming to optimize release kinetics, minimize mucosal irritation, and sustain therapeutic plasma levels. Advances in manufacturing—such as hot-melt extrusion, 3D printing, electrospinning, and spray drying—enabled enhanced control of drug release profiles, improved stability, and in some cases up to 30–50% prolongation of release time or reduction in Cmax fluctuations compared with conventional formulations. **Conclusions**: Recent formulation strategies show substantial potential to overcome long-standing limitations of SR NSAID delivery, with expected benefits for patient compliance and quality of life through reduced dosing frequency, better tolerability, and more predictable therapeutic effects. Nevertheless, integration of in vitro performance with pharmacokinetic and clinical safety outcomes remains limited, and the translation to clinical practice is still in its early stages. This review provides a comprehensive overview of current technological trends, identifies persisting gaps, and proposes future research directions to advance SR NSAID systems toward safer, more effective, and patient-focused therapy.

## 1. Introduction

Oral administration remains the most common and preferred route for drug delivery due to its convenience, non-invasiveness, cost-effectiveness, and high patient adherence. For patients and clinicians alike, the oral route offers unmatched practicality compared to injectable or transdermal systems. However, conventional immediate-release (IR) formulations are often limited by short plasma half-lives, frequent dosing requirements, and large fluctuations in systemic drug concentrations. These pharmacokinetic drawbacks can compromise therapeutic efficacy, increase the likelihood of side effects, and negatively impact patient compliance—particularly in the long-term management of chronic diseases such as arthritis, hypertension, diabetes, or persistent pain syndromes [1].

Over the past decades, modified-release (MR) oral dosage forms, including sustained-release (SR), extended-release (ER), and controlled-release (CR) systems, have emerged as key pharmaceutical strategies to address these limitations. MR technologies aim to maintain therapeutic plasma concentrations for extended periods, reduce the number of daily doses, and minimize peak–trough fluctuations, thereby supporting better clinical outcomes and improved patient quality of life [2,3,4]. These approaches are particularly relevant for polypharmacy patients, in whom regimen simplification can enhance adherence, reduce dosing errors, and potentially limit drug–drug interactions.

Among the therapeutic classes for which MR formulations are most relevant, non-steroidal anti-inflammatory drugs (NSAIDs) occupy a central position. NSAIDs remain indispensable in the management of inflammation, pain, and fever, yet their use is frequently limited by adverse effects. Gastrointestinal complications—such as dyspepsia, mucosal irritation, peptic ulcers, and gastrointestinal bleeding—arise from two distinct mechanisms: (i) Local effects, driven by the direct contact of the drug with gastric mucosa, which may be mitigated by formulation strategies such as gastro-resistant coatings, enteric polymers, or site-specific delivery systems; (ii) Systemic effects, primarily resulting from cyclooxygenase-1 (COX-1) inhibition, which remain largely unaffected by oral formulation changes and require careful drug selection and dosing to manage risk.

Other clinically significant adverse events include renal effects (e.g., sodium and water retention, reduced glomerular filtration rate, and acute kidney injury in susceptible patients), cardiovascular complications (e.g., hypertension, myocardial infarction, stroke), and, less commonly, hepatotoxicity and hypersensitivity reactions.

MR formulations—such as polymeric matrices, lipid-based carriers, microspheres, hydrogels, and multi-compartmental systems—offer opportunities to reduce the local gastrointestinal toxicity of NSAIDs by controlling drug release kinetics, limiting direct mucosal exposure, and targeting release to distal intestinal segments [5,6]. However, their potential to address systemic toxicity is inherently limited, underscoring the need for a holistic approach that combines pharmaceutical innovation with rational prescribing and monitoring.

Paracetamol, while pharmacologically distinct from NSAIDs, has been deliberately included in this review. It shares similar analgesic and antipyretic indications and faces overlapping formulation challenges—such as the need for sustained plasma levels to provide consistent pain control and the necessity of reducing peak concentrations to lower the risk of hepatotoxicity from frequent dosing [7,8]. Including paracetamol enables the identification of technological strategies that can be applied across multiple high-use analgesic agents.

In recent years, the pharmaceutical field has seen a surge in advanced formulation strategies capable of fine-tuning drug release within the gastrointestinal tract. These include mucoadhesive systems, floating dosage forms, pH-responsive polymers, enzymatically triggered matrices, electrospun nanofibers, and functionalized mesoporous carriers with customizable surface chemistry [9,10,11]. Parallel advances in manufacturing technologies—such as hot-melt extrusion (HME), spray drying, 3D printing, and electrospinning—have significantly increased the precision and flexibility of MR system design, enabling better control over release kinetics, loading efficiency, and product stability [12,13,14,15,16,17].

Despite these innovations, several gaps remain. Comparative evaluations between formulation platforms are scarce, limiting the ability to select optimal strategies for specific APIs or patient populations. Moreover, the majority of studies focus on in vitro performance, with insufficient integration of pharmacokinetic, clinical, and patient-centered endpoints. For true patient-centered therapy, formulation innovation must be aligned not only with pharmacological performance but also with the realities of adherence, comorbidity management, and polypharmacy in real-world settings.

This narrative literature review offers a formulation-focused synthesis of recent advances in extended-release oral dosage forms for NSAIDs and paracetamol. It maps technological evolution from conventional polymer matrices to hybrid and nanostructured delivery systems, analyzes the role of novel excipients and functional carriers, and highlights manufacturing approaches that enable precise control over release. By integrating technological perspectives with considerations of safety, bioavailability, and patient usability, this review aims to guide the development of oral MR systems that are not only effective and stable, but also tailored to patient needs in modern clinical practice.

## 2. Materials and Methods

A structured literature review was undertaken to identify and analyze recent developments in extended-release (ER), sustained-release (SR), controlled-release (CR), and other modified-release (MR) oral formulations of non-steroidal anti-inflammatory drugs (NSAIDs) and, where relevant, paracetamol. The purpose of this review was to examine technological strategies that modify drug release profiles, improve bioavailability, and provide more consistent pharmacokinetic control, with a focus on innovations in formulation design, excipient functionality, and advanced manufacturing methods. This work is presented as a systematic review in accordance with the PRISMA guidelines (Supplementary Materials), with a primary focus on the technological dimension of formulation development.

The literature search was conducted between February 2025 and July 2025 using the PubMed, Web of Science Core Collection, and Google Scholar databases. The search strategy combined controlled vocabulary (MeSH terms) and free-text keywords, with Boolean operators applied to maximize both specificity and coverage. Search terms included expressions such as “sustained release”, “extended release”, “controlled release”, and “modified release” in combination with “NSAID”, “nonsteroidal anti-inflammatory”, individual drug names such as “ibuprofen”, “diclofenac”, “naproxen”, “indomethacin”, “piroxicam”, “celecoxib”, “etoricoxib”, as well as “paracetamol” and “acetaminophen”, together with formulation-related terms including “oral”, “tablet”, “capsule”, and “dosage form”. The intention behind including both generic class names and individual drug names was to ensure the identification of studies that might otherwise have been missed by broader terminology alone.

**Figure 1:**
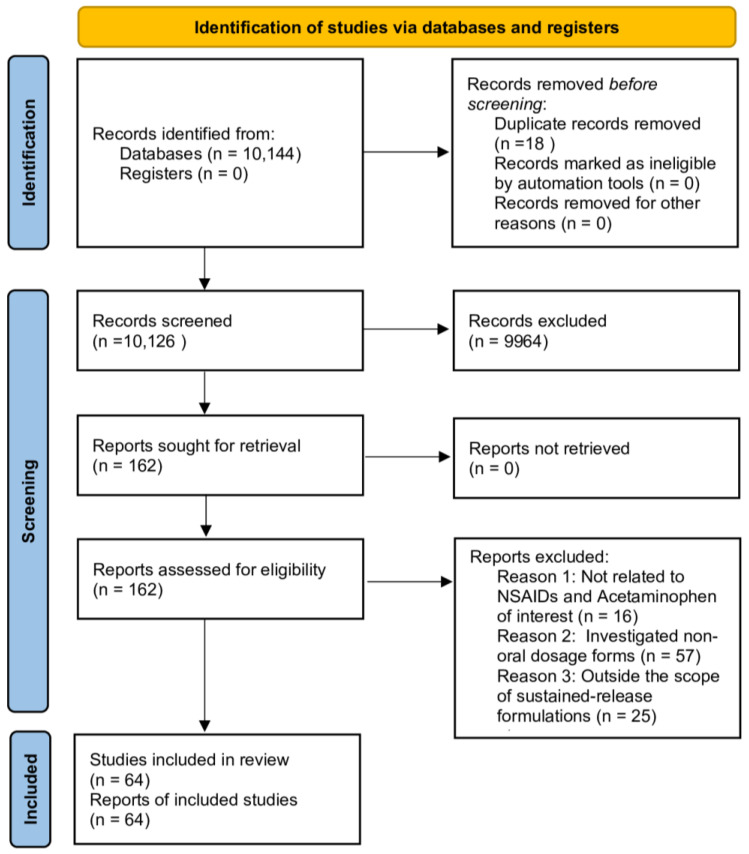
Number of publications retrieved from Web of Science Core Collection and PubMed (February–July 2025) using the search strategy described in Section 2. “Total publications” represents all hits prior to screening; “Included publications” indicates those meeting the eligibility criteria (*n* = 64).

The initial search identified 10,144 records across all databases. After removing 18 duplicates, 10,126 unique articles were screened by title and abstract. Screening was performed with a focus on the presence of relevant formulation technologies, description of the manufacturing approach, and evaluation of drug release profiles. Articles that were clearly unrelated to oral MR formulations or that lacked sufficient methodological information were excluded at this stage. This process left 162 articles for full-text review (Figure 1). During full-text evaluation, the selection was further narrowed by excluding studies that investigated only immediate-release dosage forms, non-oral administration routes, or therapeutic classes unrelated to NSAIDs or paracetamol. Studies that focused exclusively on general pharmacology without a direct link to formulation technology were also excluded.

Ultimately, 64 studies met the final inclusion criteria. These encompassed original experimental research at the in vitro, in vivo, or clinical level, as well as detailed technology-focused studies that discussed formulation composition, excipient selection, manufacturing technique, and the resulting release characteristics. Both non-selective NSAIDs and selective COX-2 inhibitors were included, as were paracetamol formulations when relevant to the objectives of the review. The inclusion of paracetamol was intentional, as it shares overlapping formulation challenges with NSAIDs, such as the need for consistent plasma concentrations and the mitigation of toxicity risk from frequent dosing.

The final set of studies represented work that provided measurable data on drug release modification, whether through achieving specific release kinetics such as zero-order or pH-dependent profiles, prolonging gastric retention, reducing burst release, or enhancing systemic bioavailability when compared with reference or immediate-release formulations. The relatively limited number of studies retained for analysis reflects the application of strict relevance and quality criteria, which excluded a substantial number of publications that, although initially retrieved by the database search, did not contain sufficient detail to allow for meaningful technological assessment. To support transparency and enable comparison between approaches, the active pharmaceutical ingredient, reference, reported formulation strategy, and main outcomes for each included study are summarized in Figure 1.

## 3. Results and Discussion

**Figure 2:**
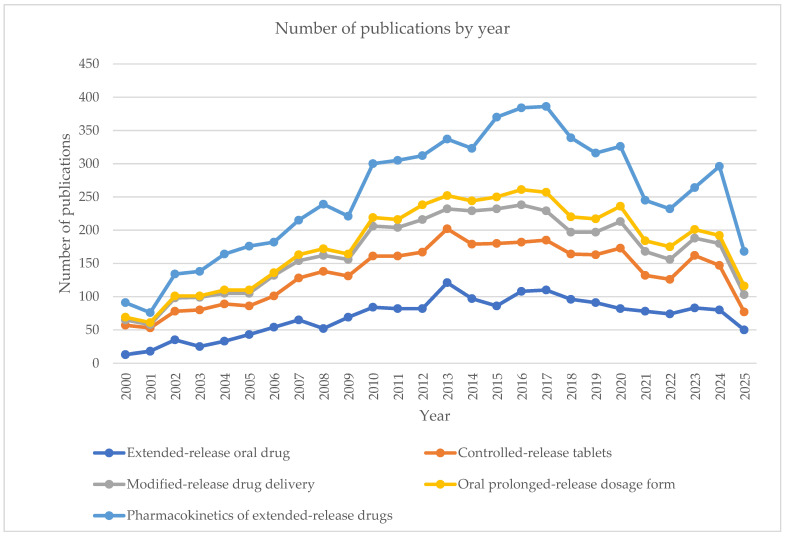
Trends in the number of publications on extended-release oral formulations of NSAIDs and paracetamol between January 2020 and July 2025, retrieved from Web of Science database using predefined search strings.

Over the past two decades, the number of scientific publications on oral modified-release drug delivery systems has steadily increased, as evidenced by data from 2000 to 2025 covering five key thematic categories: modified-release oral drug, controlled-release tablets, modified-release drug delivery, oral prolonged-release dosage form, and pharmacokinetics of modified-release drugs (Figure 2). The most significant growth has been observed in the area of modified-release oral drugs, where the number of publications rose from just 13 in 2000 to over 120 at its peak between 2013 and 2017. Simultaneously, research on the pharmacokinetics of such formulations gained momentum, particularly in the period 2015–2018. Since 2020, the growth trend has slightly slowed, potentially due to increasing specialization and a shift in focus toward emerging technologies such as 3D printing, smart drug delivery systems, and targeted therapies. This review includes the most recent data from the past five years (2020–2025), providing an up-to-date overview of current trends and innovative approaches in the development of modified-release oral formulations.

In total, sixty-one scientific publications met the predefined inclusion criteria and were subjected to full-text qualitative analysis. Each of the selected studies investigated the development and evaluation of oral modified-release dosage forms containing one or more non-steroidal anti-inflammatory drugs (NSAIDs). The primary focus of these studies was to optimize the drug release profile, improve gastrointestinal tolerability, enhance bioavailability, or prolong therapeutic activity using various formulation strategies.

Among the analyzed publications, ibuprofen emerged as the most extensively studied compound, appearing in twenty-seven independent articles. This observation highlights its dominant role as a model API in the development of modified-release drug delivery systems, likely due to its widespread use, physicochemical tractability, and well-characterized pharmacokinetics. Diclofenac sodium was the second most frequently studied drug, featured in seven publications, followed by indomethacin in six studies and paracetamol in four. Ketoprofen and aceclofenac were each addressed in three articles. Other NSAIDs, such as naproxen and flurbiprofen, were the focus of two studies each. In contrast, compounds such as etoricoxib, meloxicam, lornoxicam, celecoxib, piroxicam, aspirin, and mefenamic acid appeared only in single publications, reflecting a more exploratory stage of formulation research for these APIs in the context of prolonged-release technologies.

In terms of technological approaches, the reviewed studies encompassed a wide spectrum of formulation strategies, ranging from conventional to cutting-edge. Classical methods, such as direct compression, wet granulation, solvent casting, and ionotropic gelation, were frequently employed to produce matrix tablets, microspheres, and multiparticulate systems. Alongside these well-established techniques, a notable number of publications reported the use of advanced manufacturing platforms, including hot-melt extrusion (HME) for generating amorphous solid dispersions [18,19], electrospinning for creating nanofibrous drug delivery systems [20,21], and micro-luidic devices for precise production of lipid-based carriers [22]. Several studies explored three-dimensional (3D) printing as an emerging tool in the personalization of drug therapy, applying FDM (fused deposition modeling), DLP (digital light processing), and LCD (liquid crystal display) printing to fabricate dosage forms with tailored geometries and release kinetics.

Of particular interest were multi-component or hybrid systems that combined two pharmacologically complementary agents within a single dosage form. Examples included ibuprofen–rabeprazole combinations designed to reduce gastrointestinal toxicity through targeted drug release, and ibuprofen–carvedilol systems developed for multimodal therapeutic benefit. These studies often employed core–shell tablet architectures, layered matrices, or multiparticulate carriers to enable sequential or site-specific drug delivery.

A recurring theme across multiple publications was the strategic use of pH-responsive, mucoadhesive, and biodegradable polymers to ensure that drug release could be finely modulated by the physiological environment. Systems exhibiting pH-dependent solubility, gastric floating behavior, or colonic targeting were frequently developed to minimize systemic side effects and improve therapeutic efficacy.

The diversity of formulation techniques and release profiles reported in the analyzed studies illustrates the dynamic and multidisciplinary nature of contemporary research in modified-release NSAID delivery. From a pharmaceutical technology perspective, the choice of polymer, matrix architecture, processing method, and drug–carrier interaction critically determined the extent and mechanism of drug release. Equally important were pharmacokinetic considerations, including the achievement of sustained plasma concentrations, reduction in peak–trough fluctuations, and overall improvement in drug bioavailability.

In addition to the technological perspective, several of the reviewed systems demonstrated pharmacokinetic attributes directly translatable to clinical practice. For example, mucoadhesive formulations achieving prolonged gastric residence were associated with delayed absorption and reduced dosing frequency, while pH-dependent systems facilitated targeted drug release in the intestine, potentially lowering the incidence of upper gastrointestinal irritation. Matrix-based designs in certain studies provided near zero-order release profiles over 12–24 h, aligning with therapeutic goals of maintaining steady-state concentrations.

Although the present review does not focus on clinical trial outcomes, some included reports referenced marketed modified-release NSAID products or prototype formulations under industrial development, illustrating a clear translation pathway from formulation concept to therapeutic application. The alignment of advanced delivery systems with pharmacokinetic targets—such as sustained steady-state concentrations, minimized dosing frequency, and reduced fluctuation index—highlights their potential to improve patient adherence, reduce the burden of polypharmacy, and lower the incidence of adverse drug events. This integration of formulation science, pharmacokinetic optimization, and patient-centered design underscores the evolving role of modified-release NSAID systems in modern pharmacotherapy.

To facilitate a structured comparison of these findings, a detailed summary of all included publications is provided in Table 1. The table compiles the investigated active pharmaceutical ingredients, technological methodologies, reported formulation effects, and literature references. This comprehensive synthesis enables the identification of dominant trends, innovative solutions, and formulation–effect relationships, offering valuable insights for researchers and formulators engaged in the design of next-generation modified-release NSAID therapies.

**Figure 3:**
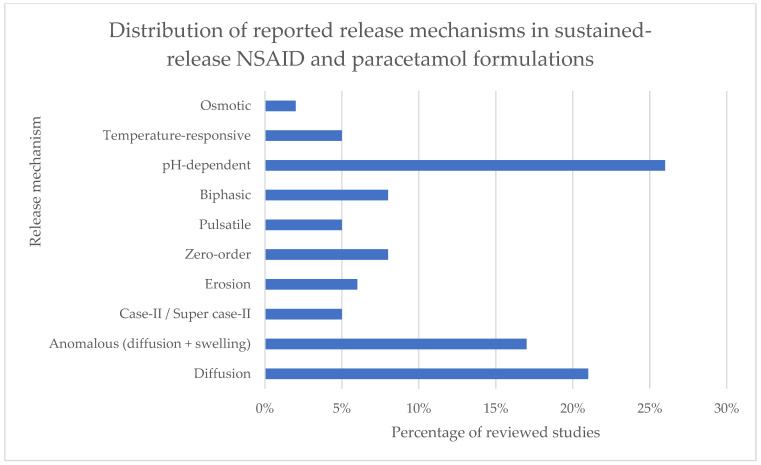
Distribution of drug release mechanisms reported in 66 studies on sustained-release formulations of NSAIDs and paracetamol. Percentages refer to the share of studies mentioning each mechanism; as some formulations combined multiple mechanisms, the total exceeds 100%.

As illustrated in Figure 3, the analysis of 66 reviewed studies demonstrates that the majority of sustained-release (SR) formulations for NSAIDs and paracetamol rely on pH-dependent systems (26%) and diffusion-controlled release (21%). This predominance is not surprising. NSAIDs are well known for causing gastric irritation, and therefore pH-sensitive coatings and carriers remain a straightforward way to protect the drug in the stomach while ensuring release in the intestine. Similarly, diffusion-controlled release, often modeled by Higuchi kinetics, represents the most classical and easiest-to-engineer approach, particularly when hydrophilic polymers (e.g., HPMC, PVP, alginate) form swelling matrices.

The third most frequent mechanism was anomalous transport (17%), combining diffusion and polymer swelling/erosion. This reflects the use of complex hydrogels and interpenetrating networks, which enable tuning of release rates by varying polymer ratios and crosslinking conditions. Such systems are attractive for academic development because they allow broad formulation flexibility without the need for specialized equipment.

By contrast, zero-order release (8%)—often considered a “gold standard” for achieving constant plasma levels—was less commonly reported. Achieving truly linear release requires more advanced design (e.g., polyelectrolyte complexes or carefully balanced bilayer matrices), which may explain its limited prevalence. Similarly, biphasic systems (8%) appear mainly where a rapid onset combined with sustained effect is clinically desirable (e.g., pain relief).

Pulsatile systems (5%) were relatively rare. Their niche use can be explained by the specific therapeutic rationale—chrono-adapted delivery for conditions such as rheumatoid arthritis, where morning stiffness is most pronounced. Such designs require more precise polymer engineering, which has not yet been widely translated into practice.

Stimuli-responsive approaches, such as temperature-responsive carriers (5%), remain at an early stage of exploration. These often rely on novel polymers (e.g., chitosan derivatives, PNIPAAm, or functionalized nanocarriers) and are mainly proof-of-concept demonstrations in vitro or in small animal models. They are promising but not yet mainstream.

Finally, osmotic pump systems (2%) were least represented. This is partly due to the high manufacturing complexity and cost associated with osmotic tablets, which restrict their application in academic research settings. Commercially, osmotic technologies have been successful in a few marketed drugs, but their complexity makes them less attractive for exploratory studies of NSAIDs.

Taken together, these data highlight a strong reliance on traditional, well-established mechanisms (pH-sensitive coatings, diffusion-based matrices), while more advanced or specialized approaches (zero-order, pulsatile, stimuli-responsive, osmotic) are still underrepresented. This reflects both practical considerations (ease of formulation, availability of excipients, regulatory familiarity) and therapeutic drivers (need for gastroprotection, reduction in dosing frequency). It also underscores the opportunity for further innovation in applying enabling technologies such as hot-melt extrusion, 3D printing, and electrospinning to achieve more sophisticated release profiles.

The prevalence of certain drug release systems in the analyzed literature reflects both their technological versatility and translational potential. For example, microspheres, hydrogel beads, and interpenetrating polymer network (IPN) systems—originally the dominant category in the previous classification—have maintained strong representation due to several practical advantages. These platforms can be tailored to achieve diverse release profiles, from zero-order to pulsatile or pH-triggered, by adjusting polymer type, cross-linking density, and particle morphology. Many utilize biocompatible and regulatory-accepted excipients such as alginate, chitosan, gelatin, and poly (lactic-co-glycolic acid), facilitating their use in oral formulations. In addition, their mucoadhesive properties enable prolonged gastrointestinal residence, enhancing drug absorption for compounds with narrow absorption windows. The high surface-to-volume ratio of microspheres further supports improved dissolution rates, particularly for poorly soluble APIs. These combined benefits make such systems attractive candidates in both exploratory formulation studies and early translational development, which explains their consistent prominence in recent research outputs.

### 3.1. Ibuprofen

Among the various formulation strategies explored for ibuprofen, a wide range of drug delivery systems has been developed to overcome its poor water solubility, short half-life, and gastrointestinal irritation potential. The most frequently reported technologies include matrix tablets, hot-melt extrusion, solid dispersions, multiparticulate systems such as microspheres and beads, and lipid-based carriers. Other emerging solutions encompass core–shell designs, 3D printing techniques, and nanostructured hybrid systems, highlighting the breadth of technological approaches aimed at optimizing the therapeutic profile of ibuprofen.

Yoon Ho et al. [22] proposed a microfluidic platform for the fabrication of monodisperse solid lipid microparticles (SLMs) composed of beeswax and Suppocire NAI 25A, used as lipid matrices to encapsulate ibuprofen, naproxen, or ROY. The microfluidic method enabled precise control over particle size and drug distribution, and ibuprofen formed a eutectic mixture with Suppocire, resulting in prolonged release for up to 72 h, which followed the Higuchi release model. This study emphasizes the potential of microfluidic-based technologies for tailoring release kinetics through rational design of lipid excipients and particle morphology.

Bulut [39] developed Fe^3+^-crosslinked semi-interpenetrating polymer network (semi-IPN) beads using sodium alginate and methylcellulose for oral ibuprofen delivery. The formulation variables such as polymer ratios and crosslinking densities were optimized to achieve high encapsulation efficiency (up to 93.3%) and modified release up to 6 h. The release followed anomalous transport kinetics, suggesting a dual mechanism involving diffusion and polymer matrix relaxation. The semi-IPN provided mechanical integrity and mucoadhesive potential, indicating its suitability for sustained gastrointestinal delivery of NSAIDs.

Ćirić et al. [40] studied the effect of drug entrapment methods on the performance of polyelectrolyte complexes (PECs) formed between chitosan and xanthan gum. Drug loading prior to PEC formation at acidic pH (4.6) with a 1:2 chitosan-to-xanthan ratio yielded the most favorable outcome, achieving near zero-order release kinetics and reduced ibuprofen crystallinity. This approach highlights the significance of pre-formulation variables and the utility of PEC systems for modulating release behavior.

Lohani et al. [10] designed pH-responsive interpenetrating polymer network (IPN) beads made from carboxymethyl konjac glucomannan and sodium carboxymethylcellulose. The beads demonstrated site-specific delivery, limiting ibuprofen release in acidic media while promoting sustained release in intestinal pH (7.4). High encapsulation efficiency, thermal stability, and structural integrity under gastrointestinal conditions make such IPN beads a viable solution to mitigate ibuprofen-induced gastric side effects.

Thadasack et al. [41] proposed embedding a dual-active pharmaceutical ingredient ionic liquid (API-IL), lidocainium–ibuprofenate, in thermoplastic zein matrices via hot-molding. INFOGEST-simulated digestion conditions showed sequential release of lidocaine and ibuprofen, where ibuprofen release was triggered at intestinal pH. The formulation preserved drug stability under thermal stress and enzymatic degradation, demonstrating the viability of API-ILs and zein as a combination for controlled oral delivery.

Khan et al. [5] developed core–shell tablets enabling sequential release of rabeprazole (gastric release) and ibuprofen (intestinal release). The system employed wet granulation with enteric-coated ibuprofen cores enclosed in an immediate-release rabeprazole shell. Eudragit L 30 D-55 provided pH-sensitive release, and the tablets exhibited good mechanical strength and long-term stability. This platform offers gastroprotection and is especially suitable for combination therapy requiring site-specific drug delivery.

Yan et al. [42] fabricated composite alginate hydrogel beads incorporating organo-modified montmorillonite (OMMT) synthesized with CTAB and NPE surfactants. These modifications increased hydrophobicity and drug affinity, resulting in enhanced ibuprofen loading and controlled release. The best-performing system released ibuprofen in a non-Fickian manner (Korsmeyer–Peppas model), with structural and morphological confirmation by FTIR, XRD, SEM, and BET [70,71]. OMMT-based beads exemplify the use of functionalized clays in matrix drug delivery systems.

Li et al. [43] developed pH-sensitive alginate aerogel beads crosslinked with Ca^2+^/Ba^2+^ ions for targeted ibuprofen delivery. Barium ions improved encapsulation efficiency and stability while minimizing swelling. The aerogel matrix suppressed release under gastric pH and enabled rapid release (96.9% within 1 h) under intestinal conditions. This dual-ion gelation technique allows the tuning of matrix responsiveness for site-specific release.

Madżarević and Ibrić [11] demonstrated the utility of visible-light-based LCD 3D printing to produce modified-release ibuprofen tablets. Compared to UV-based systems, visible-light printing achieved faster curing and higher precision. The tablets followed diffusional release profiles and retained physical integrity, confirming additive manufacturing as a scalable strategy for customized oral dosage forms.

Akaki et al. [44] introduced a remote-loading liposomal system for ibuprofen using sulfobutylether-*β*-cyclodextrin (SBE-*β*-CD) as an internal complexing agent. This suppressed the initial burst effect and provided sustained retention. The design leverages pH gradients and cyclodextrin-assisted entrapment for enhancing stability and drug loading of hydrophobic drugs, such as ibuprofen, within liposomal carriers.

Patani et al. [45] assessed the performance of Irvingia gabonensis polymer as a matrix former in direct-compression ibuprofen tablets, in comparison to HPMC and xanthan gum. The Irvingia-based tablets exhibited robust mechanical strength and controlled release governed by Korsmeyer–Peppas kinetics. Different drying methods influenced polymer swelling and release rates. This study underlines the potential of natural excipients for cost-effective, sustained-release formulations.

Yousefi et al. [46] synthesized a nanocomposite carrier composed of magnetic Fe_3_O_4_ cores coated with layered double hydroxides (LDH), intercalated with ibuprofen and diclofenac. Drug release followed a sustained profile under pH 7.4, and the nanostructure exhibited structural stability and magnetic responsiveness. The system allows co-delivery and targeted release, applicable to anti-inflammatory therapy in localized conditions.

Samuel et al. [47] designed an asymmetric membrane floating nanoparticle (AMFNP) system for ibuprofen using a phase inversion technique. The nanoparticles demonstrated prolonged gastric residence, improved solubility, and enhanced anti-inflammatory efficacy. Drug release followed Higuchi kinetics and was governed by Fickian diffusion. This platform offers a gastroretentive solution to improve ibuprofen bioavailability and minimize side effects.

Choi et al. [48] developed dual-responsive mesoporous silica nanoparticles (RMSNs) encapsulated in thermo-sensitive agarose gels for ibuprofen delivery. The system enabled pH- and temperature-triggered release and exhibited a remarkable drug-loading capacity (~270 wt%). The nanoparticles were biocompatible and highly responsive to simulated physiological conditions, supporting their applicability in intelligent oral drug delivery.

Zarinwall et al. [50] evaluated mesoporous silica aerogels (SA) functionalized for enhanced ibuprofen solubilization and amorphization. Post-synthetic solvent-free loading (via co-milling or melting) led to improved dissolution and stability. The release profile depended on surface hydrophilicity, with both hydrophobic and hydrophilic carriers providing modified release. The results confirm SA as a robust carrier for poorly soluble drugs.

Varghese et al. [51] proposed an iron-based nano-biocomposite (Fe-CNB) embedded in alginate hydrogels for colon-specific ibuprofen delivery. The pH-responsive system achieved targeted release in colonic pH while minimizing gastric release. Cyclodextrin and Fe-CNB synergy enhanced encapsulation and release modulation. This approach is particularly suited for inflammatory bowel diseases and site-specific therapy.

Chen et al. [19] formulated sustained-release amorphous solid dispersions (ASDs) using a binary matrix of hydrophilic PVP VA64 and hydrophobic RSPO via hot-melt extrusion. The optimized ASD displayed a consistent release plateau and enhanced stability. Intermolecular interactions between matrix components ensured homogeneous drug distribution. This scalable platform illustrates the advantages of rational polymer blending for sustained-release dosage forms.

Albarahmieh et al. [52] utilized a natural polymer blend (cellulose acetate butyrate and colophony) to formulate ibuprofen ASDs via spin coating and HME. Spin-coated systems offered greater stability, while HME-based formulations provided modified release over 5 h. Both systems followed first-order kinetics, supporting the potential of bio-based polymers in modified-release systems.

Akin-Ajani et al. [53] evaluated Talinum triangulare-derived polymers (mucilage and low-methoxyl pectin) in microsphere formulations of ibuprofen. Ionotropic gelation produced formulations with sustained or immediate release depending on polymer ratios. The natural polymers showed promising encapsulation efficiency (~ 60%) and desirable swelling behavior, confirming their utility in controlled-release delivery.

Che et al. [20] introduced a dual-jet electrospinning approach to produce ibuprofen-loaded nanofibers with both immediate- and sustained-release layers. The nanofibers exhibited high drug amorphization, reduced recrystallization risk, and minimal gastric irritation. This strategy enables one-step fabrication of dual-phase delivery systems with improved tolerability.

Uddin et al. [54] developed amorphous solid dispersions of ibuprofen using melt fusion and freeze-drying, subsequently compressed into sustained-release tablets. Both techniques enhanced solubility and provided 12 h drug release with Weibull kinetics. While physicochemical properties varied between methods, dissolution performance remained consistent, indicating the robustness of the ASD approach for prolonged ibuprofen delivery.

Collectively, the reviewed technologies illustrate a broad spectrum of formulation innovations tailored to address the specific biopharmaceutical limitations of ibuprofen. From polymeric networks and ionotropic gels to 3D-printed dosage forms and lipid nanocarriers, the continual evolution of delivery systems enables customization of release profiles, enhancement of solubility, and mitigation of gastrointestinal side effects. The integration of emerging technologies, such as additive manufacturing and responsive nanomaterials, positions ibuprofen as a model API for exploring and refining next-generation drug delivery platforms.

### 3.2. Flurbiprofen

Bulut et al. developed interpenetrating polymer network (IPN) beads composed of sodium alginate, polyvinyl alcohol, and methylcellulose for the controlled release of flurbiprofen (FBP) [36]. The beads were crosslinked with glutaraldehyde and characterized using FTIR, DSC, and SEM techniques. Drug release behavior was modulated by polymer composition, crosslinking time, and drug-to-polymer ratio. The optimal formulation (NaAlg/PVA/MC 4:1:1, FBP/polymer 1:4, crosslinking for 15–30 min) showed the highest release of FBP over 6 h, with non-Fickian release kinetics. This study demonstrates the potential of water-soluble polymer-based IPNs for tailoring the release of NSAIDs such as flurbiprofen.

Işıklan and Erol designed temperature-responsive nanospheres based on chitosan and hydroxypropyl cellulose (CS/HPC) for controlled delivery of flurbiprofen [37]. Nanocarriers were obtained via the emulsion method and characterized using ATR-FTIR, XRD, SEM, DSC/TGA, zeta potential, and particle size analysis. The nanospheres displayed a lower critical solution temperature (LCST) of 42 °C. In vitro release studies at 30 °C, 37 °C, and 44 °C revealed temperature-dependent drug release, with reduced release at elevated temperatures, indicating a pore-closing mechanism. The CS/HPC ratio, drug loading, and crosslinker concentration all influenced release kinetics. Cytotoxicity assays confirmed biocompatibility, suggesting suitability for temperature-sensitive delivery of anti-inflammatory agents.

Erol et al. further explored the potential of chitosan–graphene oxide (CS-GO) blend nanoparticles for flurbiprofen delivery [38]. Nanoparticles were prepared by emulsion method and characterized with FTIR, DSC, TGA, XRD, SEM, and AFM. The introduction of graphene oxide enhanced thermal stability. Average particle sizes ranged from 362 to 718 nm with zeta potential values between −7.67 and −27.93 mV. In vitro studies revealed biphasic release with an initial burst followed by sustained release. The GO content modulated release kinetics, and cytotoxicity studies confirmed their biocompatibility, positioning CS-GO nanoparticles as promising carriers for NSAID delivery.

### 3.3. Ketoprofen

García et al. compared the dissolution behavior of enteric-coated (EC) and modified-release (XR) ketoprofen formulations under simulated intestinal conditions using low-molarity phosphate buffers [14]. EC formulations demonstrated sensitivity to polymer composition, correlating well with in vivo performance. However, XR formulations displayed variability due to excipients such as dibasic calcium phosphate, with in vivo data showing improved buffering equilibrium. Their findings advocate for testing XR ketoprofen in higher-molarity buffers to ensure biopredictive dissolution profiles.

Shamim et al. formulated sustained-release matrix tablets (MTs) of ketoprofen using surfactant-assisted wet granulation (SAWG) without employing specialized excipients [60]. The optimized formulation (MT2) with 3% Soluplus^®^ exhibited a swellable-erodible profile and sustained release over 24 h. In vivo studies confirmed delayed Tmax, reduced Cmax, and maintenance of plasma levels above the minimum effective concentration for 24 h. MT2 achieved a 2.3-fold higher AUC compared to the non-surfactant control, demonstrating improved pharmacokinetic performance.

Pyteraf et al. demonstrated the use of a single hot-melt extruded filament of poly (vinyl alcohol) and ketoprofen to fabricate 3D printed tablets with immediate, sustained, and layered release profiles [59]. Modifications in tablet geometry, infill density, and internal architecture enabled varied drug release, without altering filament composition. Tablets retained amorphous ketoprofen and reproducible structure, supporting the versatility of FDM-based 3D printing for personalized NSAID delivery.

Vo et al. developed delayed-release ketoprofen pellets using continuous hot-melt extrusion with real-time monitoring via inline NIR and pellet size analysis [61]. A full factorial design optimized stearic acid content, drug load, and pellet size. The optimized formulation released <5% in SGF after 120 min and >95% in SIF within 45 min. This continuous process enabled precise manufacturing and efficient gastric resistance.

### 3.4. Loxoprofen

Anam et al. designed mucoadhesive sustained-release microspheres using pectin (PEC) and its thiolated derivative (T-PEC) for loxoprofen delivery [72]. Thiolated pectin was synthesized via esterification with thioglycolic acid, confirmed by FTIR and –SH content. Microspheres prepared by solvent evaporation exhibited favorable mucoadhesion, spherical morphology (2–10 μm), and sustained release (>80% over 12 h). In vivo and ex vivo studies confirmed prolonged mucosal retention and anti-inflammatory efficacy for up to 24 h, supporting the therapeutic potential of thiolated pectin-based systems in arthritis.

### 3.5. Naproxen

Freitas et al. developed naproxen-loaded beads based on sericin/alginate blends for delayed and sustained release [66]. Among various crosslinking strategies, the non-crosslinked formulation achieved highest entrapment (>80%) and prolonged release (~360 min), with minimal acidic release. Characterization confirmed polymer-drug compatibility and thermal stability. This multiparticulate system demonstrates suitability for pH-dependent NSAID delivery.

Hameed et al. fabricated polymer hybrid enteric microspheres (PHE-Ms) combining Eudragit L100 and HPMC-E5 for naproxen delivery [67]. Microspheres exhibited particle sizes of ~29–74 μm, low Span index (0.49–0.69), and high entrapment efficiency. Structural analysis showed reduced crystallinity. Formulation effectively limited acidic release and enabled controlled intestinal drug release.

### 3.6. Aceclofenac

Rashid et al. engineered pulsatile-release aceclofenac tablets using press-coated technology [23]. Immediate-release core tablets were coated with combinations of HPMC K100M, Eudragit L100, and HEC to match circadian rhythms in rheumatoid arthritis. One formulation achieved 99% release at 6 h with minimal release at earlier time points, supporting chronotherapeutic efficacy.

Ibrahim et al. prepared sustained-release aceclofenac matrix pellets using extrusion-spheronization with Eudragit RL100 and polyvinylpyrrolidone [24]. Response surface methodology optimized excipient ratios. The optimized system provided limited acidic release and prolonged alkaline release, indicating potential for once-daily administration.

### 3.7. Diclofenac

Viscusi and Gorrasi developed alginate-based beads encapsulating LDH intercalated with diclofenac sodium [31]. LDH enhanced bead thermal stability and conferred pH- and temperature-responsive release. Release profiles correlated with environmental conditions, suggesting suitability for triggered NSAID delivery.

Sarkar et al. optimized modified-release diclofenac beads using pectin and taro stolon polysaccharide (TSP), crosslinked with CaCl_2_ [5]. The optimized formulation showed high DEE (88.5%), sustained release (T90 = 11.4 h), and favorable release similarity factor (f2 = 71.6), indicating its utility in polymeric matrices.

Obeidat et al. studied compaction behavior of directly compressed diclofenac tablets using Kollidon SR and excipients like MCC and PVA100 [32]. The optimized quaternary formulation showed robust mechanical properties and release kinetics fitting the Korsmeyer-Peppas model, confirming its controlled-release potential.

Sanoufi et al. employed DoE-based optimization to formulate modified-release diclofenac via hot-melt extrusion [18]. A D-optimal design yielded predictable drug release profiles with high R^2^ values and confirmed the robustness of formulation variables.

Nguyen et al. prepared silk fibroin nanoparticles functionalized with PVP K30 for diclofenac delivery using solvent exchange and adsorption [33]. Nanoparticles showed ~40% encapsulation, acid-resistant behavior, and sustained intestinal release. In vivo studies demonstrated enhanced anti-inflammatory efficacy.

Silva et al. created cashew gum nanoparticles grafted with PPG for sustained diclofenac delivery [34]. The system featured spherical morphology, a size of ~275 nm, and prolonged release up to 68 h, confirming thermal and release stability.

Crișan et al. fabricated bilayer diclofenac tablets using FDM and HME [29]. A fast-release honeycomb layer and a sustained-release filled layer enabled dual-phase release. Polyvinyl alcohol supported both layers, exemplifying 3D printing’s potential for personalized drug delivery.

### 3.8. Indomethacin

Gong et al. developed a composite fiber made of sodium alginate and feather keratin with a distinctive core–shell architecture using a wet-spinning method, aiming to design a carrier system for sustained indomethacin release [55]. FTIR spectroscopy confirmed interactions among sodium alginate, feather keratin, and indomethacin, while UV–Vis spectrophotometry was used to monitor the drug release profiles in simulated gastric, intestinal, and colonic fluids. SEM analysis revealed the fiber’s morphology. Drug release was found to be pH-dependent: less than 20% of the drug was released in gastric fluid, while nearly 80% was released within 12 h in both intestinal and colonic media. Increasing the proportion of feather keratin in the formulation further prolonged the release of indomethacin, highlighting its potential to reduce gastric side effects and enable intestinal targeting.

Das Karmakar and Pal synthesized an amphiphilic dextran-based copolymer via RAFT polymerization using a novel hydrophobic methacrylic monomer [56]. The resulting polymer exhibited controlled molecular weight, low dispersity, and self-assembled into micelles in aqueous solutions. These micelles demonstrated high encapsulation efficiency for indomethacin and facilitated sustained drug release. Electron microscopy confirmed the nanostructure, and MTT assays showed the formulation was non-toxic up to 100 µg/mL. This dextran-based system offers a promising platform for poorly soluble drugs requiring controlled delivery.

Vieira et al. formulated mucoadhesive beads by blending κ-carrageenan and sericin, aimed at modified oral delivery of indomethacin [16]. These beads demonstrated pH-dependent swelling (maximal at pH 6.8) and strong mucoadhesive properties. Thermal analysis confirmed the structural stability, while no chemical interactions with the drug were observed. Biocompatibility was demonstrated using 2D and triple-culture models with >70% cell viability. Although the permeation of encapsulated indomethacin (6.3%) was lower than free drug (10.9%), the system improved mucosal contact time and prolonged release, making it suitable for safer oral administration.

Esim et al. designed mucoadhesive buccal tablets containing indomethacin, focusing on the impact of polymers (chitosan, carbopol, HPMC) and diluents (mannitol, lactose, MCC) on swelling, mucoadhesion, and release behavior [17]. A 3^2^ full factorial design was used to assess formulation effects. Tablets with higher chitosan or carbopol levels had slower drug release and higher swelling indices (*p* < 0.05). Drug release in simulated saliva followed anomalous transport kinetics (Case II and Super Case II), suggesting that both swelling and diffusion mechanisms played roles. Mannitol-enhanced dissolution, and the hydrated tablets formed cohesive gel matrices. The study underlined the influence of excipient selection on buccal drug delivery.

Al-Hashimi et al. developed orodispersible tablets incorporating indomethacin-loaded pellets coated with Eudragit L100 to achieve delayed release [57]. The pellets, prepared by extrusion–spheronization using different Eudragit particle sizes, were embedded into tablets. The 63 µm formulation offered optimal compressibility and release performance. These tablets disintegrated rapidly (14 ± 0.6 s) but maintained pellet integrity, showing minimal release at pH 1.2 and fast drug release at pH 6.8. This design improved patient compliance and reduced gastric irritation.

Damiati and Damiati developed a novel platform combining microfluidics and machine learning to optimize the synthesis of indomethacin-loaded PLGA microparticles [58]. Using a 3D flow-focusing chip, the authors generated monodisperse PLGA droplets and trained an artificial neural network (ANN) to predict particle size based on polymer concentration and flow rates. Optimized conditions yielded uniform microparticles with good encapsulation efficiency (EE ~62%) and drug loading (~7.8%). In vitro studies demonstrated a biphasic, sustained release reaching ~80% over nine days. This work highlights the potential of integrating AI-driven prediction with microfluidics to produce size-tunable, reproducible polymeric carriers for poorly soluble drugs like indomethacin.

### 3.9. Ketorolac

Naeem et al. developed pH-sensitive hydrogels composed of chondroitin sulfate and Pluronic F-127 for the controlled release of ketorolac tromethamine, a potent NSAID with a short half-life [62]. Synthesized via free radical polymerization, the hydrogels used acrylic acid as a monomer, N,N’-methylene bisacrylamide as a cross-linker, ammonium persulfate as an initiator, and Tween-80 as a surfactant. These blends exhibited enhanced swelling and water uptake at pH 7.4, with in vitro release following zero-order kinetics over 36 h. Structural characterization (FTIR, SEM, thermal analysis, and XRD) confirmed network integrity and stability. Toxicity studies in rabbits indicated excellent biocompatibility. The study proposed this hydrogel as a promising delivery platform for reducing ketorolac dosing frequency and improving therapeutic consistency.

Aldawsari et al. developed pulsatile compression-coated tablets of ketorolac tromethamine tailored for chronotherapeutic release, particularly targeting morning arthritis symptoms [12]. The design included a fast-disintegrating core and an outer coating made of polyethylene oxide (PEO) and Eudragit RLPO to control lag time. A central composite experimental design optimized the formulation variables, including polymer concentrations and tablet hardness. The final formulation achieved a 9-h lag time followed by complete release within 17.42 h. In vivo imaging and pharmacokinetics confirmed effective delayed release and alignment with circadian pain patterns. This system demonstrates the potential of pulsatile platforms for optimized therapeutic outcomes in chronobiology-driven diseases.

### 3.10. Paracetamol

Đuranović et al. employed fused deposition modeling (FDM) 3D printing to prepare modified-release paracetamol tablets from hot-melt extruded filaments composed of polyethylene oxide (PEO), polycaprolactone (PCL), arabic gum, and Gelucire^®^ 44/14 (17). Formulations achieved up to 60% *w/w* drug load. Mechanical testing assessed filament printability, while a decision tree model predicted success with 84.85% accuracy. PEO-based filaments enabled faster drug release but caused more printhead clogging, while PCL-based filaments exhibited slower, sustained release. Drug release ranged from 50% over 8 h (PCL) to complete in 4 h (PEO), with mechanisms combining diffusion and erosion.

Enke et al. introduced 3D screen printing (3DSP) to fabricate customizable paracetamol dosage forms with tailored release kinetics [8]. Immediate-release (IR) and modified-release (ER) pastes were used separately or in combination to produce disk- and donut-shaped tablets. The resulting printlets met Ph. Eur. standards for size, mass, friability, and breaking strength. Release testing confirmed tunability via geometrical and compositional adjustments. This work highlighted the precision and adaptability of 3DSP for individualized oral drug delivery systems.

Poortinga et al. proposed a novel microbubble-based encapsulation technique for paracetamol, targeting both taste-masking and controlled enteric release [68]. Micronized paracetamol was dispersed in cyclohexane with hydrophobized silica, emulsified with maltodextrin, and freeze-dried to yield gas-in-liquid microbubbles. Encapsulation efficiency exceeded 90%, and sensory evaluation confirmed effective taste-masking. The formulation showed minimal release in saliva and gastric fluids but rapid, complete release in simulated intestinal fluid containing bile salts. This technology presents a novel strategy for pediatric and geriatric liquid dosage forms.

Pishnamazi et al. utilized amine-functionalized mesoporous silica (KCC-1-NH_2_) as a novel carrier for paracetamol in controlled-release tablets [6]. KCC-1 was synthesized hydrothermally and modified with APTES. Paracetamol was loaded via solvent evaporation, and tablets were compressed directly. Characterization (FTIR, SEM, TEM, N_2_ sorption) confirmed successful functionalization and structural stability. In vitro studies demonstrated that KCC-1-NH_2_ significantly prolonged drug release compared to KCC-1 and MCC controls. The findings confirm that chemical modification of mesoporous silica can effectively tailor release kinetics, offering promise for precision oral delivery platforms.

### 3.11. Other NSAID Ingredients

Xu et al. developed dual-component enteric-coated pellets containing aspirin and L-glutamate, aiming to simultaneously deliver an anti-inflammatory agent and a gastroprotective amino acid [25]. The formulation employed extrusion–spheronization for core preparation and fluidized bed coating for enteric protection. The design ensured separate cores for each active pharmaceutical ingredient, followed by coating with pH-sensitive polymers to achieve a two-phase release profile. Physicochemical evaluations, including salicylic acid quantification, dissolution profiling, and morphological analysis, confirmed the product’s stability and performance, indicating its promise in minimizing gastrointestinal damage commonly associated with aspirin use.

Alhajj et al. developed aspirin-loaded nanoparticles using the solvent evaporation method with sodium alginate and PVP as stabilizers. [26] The formulation yielded spherical particles (76–128 nm, ζ +36 to +48 mV) with moderate encapsulation efficiency (33–44%) and good stability. In vitro release followed diffusion/anomalous transport, reaching ~97% within 24 h, while antioxidant assays and an in vivo thrombosis model confirmed enhanced pharmacological activity compared to pure aspirin, indicating the potential of this system to improve safety and efficacy.

Biji et al. formulated mucoadhesive microbeads composed of sodium alginate and carboxymethyl chitosan (CMC) for the sustained intestinal release of celecoxib, targeting inflammatory bowel diseases [27]. The beads were produced via ionic gelation and optimized using response surface methodology. The optimized system demonstrated strong mucoadhesion (~59%) and sustained drug release over 24 h. Cytotoxicity testing confirmed non-toxicity in the 100–250 µM range, while anti-inflammatory activity was evidenced by a 61% reduction in nitric oxide levels in LPS-stimulated HCT-116 cells. Additional in vitro assessments showed suppressed reactive oxygen species production and cyclooxygenase-2 expression, supporting the microbeads’ efficacy as a biocompatible and sustained-release celecoxib delivery platform for intestinal inflammation management.

Mudhakir et al. designed dual-functionalized mesoporous silica nanoparticles (MSNs) for the pH-responsive delivery of celecoxib [22]. MSNs were synthesized via the sol–gel method and grafted with aminopropyl silane to enhance drug loading capacity (12.91%) and enable conjugation with imidazole-functionalized polyethyleneimine (PEI) as a pH-sensitive gatekeeper. Drug release was significantly increased under acidic conditions (pH 5.5), with a 33% improvement in release within 2 h. Cytotoxicity testing in RAW 264.7 macrophages revealed lower toxicity for the functionalized carriers compared to unmodified PEI, suggesting potential for targeted anti-inflammatory and anticancer applications with reduced systemic exposure.

Batool et al. developed chitosan/guar gum-based celecoxib hydrogel beads using ionotropic gelation to create both single and dual crosslinked structures [29]. The formulations achieved entrapment efficiencies of ~55% and ~44% for single and dual crosslinked beads, respectively. The dual crosslinked system demonstrated enhanced mucoadhesiveness and slower release (~24% over 24 h) compared to the single crosslinked variant (~74%). In vivo evaluation in rats revealed significant anti-inflammatory activity, with reductions in paw edema and systemic markers (CRP and IL-6), supporting the hydrogel beads’ therapeutic potential in sustained NSAID delivery.

Sun et al. developed nano-lyophilized orally disintegrating tablets (ODTs) of celecoxib using a combination of media milling and freeze-drying to enhance solubility and oral bioavailability [30]. The optimized formulation contained 49.5% celecoxib with PVP K30, SDS, and mannitol as stabilizers and cryoprotectants, producing nanocrystals with an average size of ~351 nm. The tablets disintegrated within 5 s and released more than 90% of the drug within 3 min across all tested pH values. In vivo studies in rats and dogs demonstrated markedly improved bioavailability (155% and 292% vs. Celebrex^®^, respectively) and reduced Tmax, highlighting the potential of this nanotechnology-based ODT platform to lower therapeutic doses while maintaining efficacy.

Saady et al. developed gastro-floating bilayer tablets for the dual sustained release of etoricoxib and famotidine, using natural and semi-synthetic swellable polymers such as konjac gum, guar gum, xanthan gum, and HPMC K15M [15]. A full factorial design optimized the formulation, which exhibited rapid buoyancy (floating lag time: ~50 s), high swelling index (297.7%), and modified drug release for both APIs over 8 h. In vivo pharmacokinetics showed approximately 2-fold increased AUC values for both drugs compared to commercial tablets, with relative bioavailabilities exceeding 200%. These results confirm the formulation’s suitability for gastroretentive dual-drug delivery, improving patient compliance and reducing NSAID-associated gastric risks.

Vieira et al. engineered thermally and covalently crosslinked beads composed of κ-carrageenan and sericin for modified-release delivery of mefenamic acid [64]. The beads achieved high drug entrapment (94.11–104.25%) and loading capacities (36.50–47.50%). Drug release kinetics fit the Weibull model and involved diffusion, matrix relaxation, and erosion mechanisms. Thermal analysis (DSC, TGA) and FTIR confirmed the compatibility and stability of the drug–polymer matrix. Cell viability assays demonstrated improved biocompatibility of the encapsulated formulation compared to free mefenamic acid, suggesting a favorable safety profile for chronic administration.

Tung et al. introduced a biphasic tablet for lornoxicam, integrating fast-release nanocrystals in an immediate-release layer and a sustained-release HPMC-based matrix core [63]. Lornoxicam nanocrystals were prepared using a top-down technique combining jet and ball milling, yielding nanoparticles (~280 nm) stabilized by PVP K30. The compression-coated tablets released the drug in two phases, with the formulation optimized via Design of Experiments. This hybrid system enhances therapeutic flexibility and is especially suied for conditions requiring both rapid and prolonged drug action.

Navarro-Ruíz et al. designed multiparticulate Eudragit-based systems for colon-targeted meloxicam delivery [65]. Formulations containing Eudragit NM and cellulose exhibited optimal pH-dependent release, delaying drug liberation until reaching colonic pH (6.0–7.0). The release mechanism followed Higuchi and first-order kinetics, indicating a combined diffusion and swelling–erosion model. This colonic-targeted strategy may improve therapeutic outcomes in colorectal diseases, such as cancer and autoimmune conditions.

Friuli et al. incorporated electrospun nanofibers of meloxicam and carvedilol into tablet matrices for pH-independent controlled oral delivery [62]. The nanofibers, produced via electrospinning, significantly enhanced solubility and modulated release profiles, with kinetics fitting various models including Higuchi and Korsmeyer–Peppas. The resulting tablets achieved both immediate and sustained-release effects, showcasing electrospinning as a versatile platform for tailoring oral drug release across diverse gastrointestinal environments.

Finally, Pham et al. formulated nanostructured lipid carriers (NLCs) for the oral delivery of S-(+)-zaltoprofen to address its low bioavailability [69]. The system, optimized via Box–Behnken design, demonstrated particle sizes around 105 nm, encapsulation efficiency > 99%, sustained release, and enhanced intestinal permeability. Pharmacokinetic studies in rats revealed a 4.3-fold increase in relative oral bioavailability compared to the unformulated drug, confirming the feasibility of NLCs as carriers for poorly soluble NSAIDs.

### 3.12. Future Development Strategies

Based on the analysis of the literature from 2020 to 2025 and the identified technological trends in sustained-release oral formulations for NSAIDs, several research directions emerge that may guide the future development of this field. These strategies combine insights from pharmaceutical technology, pharmacokinetic optimization, and patient-centered therapy, aiming to address current limitations while anticipating clinical and industrial needs.

Future research in the field of sustained-release oral formulations for NSAIDs should follow a multi-dimensional approach, integrating technological innovation with pharmacokinetic optimization and patient-centered design. One promising direction lies in the development of next-generation biodegradable and biocompatible polymers, particularly hybrid systems that combine pH-dependent release with mucoadhesive properties. Such materials can be engineered to fine-tune drug release in specific regions of the gastrointestinal tract, responding both to luminal pH and gastrointestinal motility patterns. This dual-responsiveness may improve site-specific targeting and minimize systemic side effects, enhancing therapeutic efficacy.

Another important avenue involves the integration of smart and responsive elements into oral dosage forms. Although still at an early stage for oral delivery, the incorporation of sensing or stimuli-responsive components—capable of adjusting drug release in response to physiological cues such as inflammatory biomarkers, temperature fluctuations, or enzymatic activity—could enable real-time modulation of therapy. This approach may reduce the risk of under- or overdosing in fluctuating disease states, aligning drug release more closely with the patient’s dynamic clinical condition.

Equally critical is the focus on scalability and industrial translation. Techniques such as hot-melt extrusion (HME) and 3D printing with pharmaceutical-grade polymers hold significant potential for bridging the gap between laboratory-scale prototypes and commercial products. Research in this area should not only demonstrate the therapeutic performance of such systems but also address the development of robust process analytical technologies (PAT) and regulatory-compliant quality control strategies, ensuring reproducibility and quality at manufacturing scale.

Finally, the growing field of personalized medicine offers an opportunity to adapt sustained-release NSAID formulations to patient-specific needs. By integrating advances in digital health, therapeutic drug monitoring, and real-time adherence tracking, it may become possible to design formulations with individualized dosing regimens, optimizing both safety and efficacy. Such convergence of formulation science, industrial feasibility, and patient-tailored therapy could define the next generation of sustained-release NSAID delivery systems, ensuring their clinical relevance and long-term impact.

## 4. Conclusions

The landscape of oral drug delivery is undergoing a transformative evolution, driven by the pressing need to optimize therapeutic efficacy, minimize adverse effects, and enhance patient adherence—particularly in the context of chronic diseases requiring long-term pharmacological management. Modified-release (MR) oral formulations, encompassing sustained-, delayed-, and targeted-release systems, have emerged as a cornerstone in modern pharmaceutical development. As demonstrated by the recent surge in innovative formulation strategies, it is now possible to precisely modulate the release kinetics of both conventional and challenging active pharmaceutical ingredients (APIs), including poorly soluble drugs and those with narrow therapeutic windows.

This review highlights a broad spectrum of technological advancements and formulation approaches that have significantly expanded the capabilities of MR dosage forms. The incorporation of hydrophilic matrices, mucoadhesive polymers, nanostructured carriers, and biodegradable or pH-responsive excipients has enabled the development of systems tailored to the physicochemical properties of the API and the physiological conditions of the gastrointestinal tract. Notably, the evolution of multiparticulate drug delivery systems—such as coated pellets, electrospun nanofibers, and lipid-based carriers—has allowed for fine-tuned, site-specific release and dual-drug delivery options, offering new solutions for combination therapy and polypharmacy.

Emerging technologies such as hot-melt extrusion (HME), 3D printing, mesoporous silica functionalization, and advanced crosslinking networks further support the design of highly customizable and scalable MR formulations. The successful implementation of these approaches, supported by robust in vitro and in vivo performance, demonstrates that modern formulation science can overcome classical barriers such as first-pass metabolism, fluctuating plasma levels, and dose dumping. Moreover, the integration of Quality by Design (QbD), in-line process monitoring (e.g., NIR), and modeling tools ensures that these innovations are not only effective but also industrially viable and regulatory compliant.

Among these emerging methods, HME has become particularly prominent due to its versatility and industrial relevance. Its key advantages include continuous processing, the generation of solid dispersions, and the ability to enhance the solubility of poorly water-soluble active pharmaceutical ingredients (APIs). These properties are especially important for non-steroidal anti-inflammatory drugs (NSAIDs), which are often limited by poor aqueous solubility and erratic absorption. Embedding NSAIDs into polymeric carriers through HME enables improved dissolution, more reproducible release profiles, and enhanced bioavailability.

Another major benefit of HME is its compatibility with a wide range of functional excipients, allowing precise tuning of release kinetics. Hydrophobic polymers such as ethylcellulose can extend release for up to 24 h, while hydrophilic polymers (e.g., PVP VA64, Soluplus^®^) balance hydration, swelling, and erosion dynamics to achieve near-constant release rates. HME has also been used to design biphasic formulations, combining rapid onset with sustained effects—an attractive profile for analgesics such as ibuprofen or diclofenac.

Recent advances have further expanded the role of HME beyond conventional matrices. Between 2022 and 2024, studies reported HME-based 3D printing filaments, thin films, and hybrid extrusion–deposition systems for personalized drug delivery. These developments demonstrate that HME is not only a mature manufacturing process but also a bridge to digital and patient-tailored technologies. Importantly, HME is already recognized by regulatory agencies as a scalable, reproducible, and cGMP-compliant process, which strengthens its translational potential for clinical applications.

Finally, the reviewed literature underscores the broader clinical relevance of MR platforms. Formulations incorporating NSAIDs such as celecoxib, etoricoxib, zaltoprofen, and meloxicam illustrate how MR strategies can mitigate gastrointestinal toxicity, reduce systemic exposure, and sustain pharmacodynamic effects. Systems that integrate immediate- and sustained-release components—such as biphasic tablets and compression-coated platforms—further exemplify the shift toward personalized pharmacotherapy, where timing and site-specific drug release are optimized for therapeutic benefit.

Despite these advances, challenges remain. Scale-up feasibility, long-term physical and chemical stability, excipient compatibility, and inter-patient variability must be rigorously addressed through continued research. Additionally, as drug delivery systems become more complex, multidisciplinary collaboration among formulation scientists, pharmacologists, materials engineers, and regulatory experts will be essential to ensure safe and effective translation from bench to bedside.

Beyond their technological attributes, modified-release NSAID systems have a direct connection to patient-centered therapy principles. By reducing dosing frequency and providing more consistent plasma drug levels, such formulations can enhance adherence, particularly in chronic conditions where treatment regimens are often complex. This is of particular value in polypharmacy, where minimizing the number of daily doses and reducing peak–trough fluctuations can help decrease the risk of drug–drug interactions and simplify medication schedules. From an industry perspective, translating advanced formulation concepts into commercial products presents several challenges, including process scale-up, batch-to-batch reproducibility, cost-effectiveness of raw materials, and compliance with evolving regulatory requirements. Furthermore, bridging the gap between promising in vitro or preclinical performance and consistent clinical outcomes remains a key translational hurdle. Addressing these factors is essential for ensuring that the benefits of advanced modified-release NSAID systems can be realized in real-world patient care.

In summary, recent advances in the design and optimization of modified-release NSAID formulations demonstrate significant progress in achieving sustained therapeutic effects, improving patient adherence, and reducing adverse event incidence. These innovations reflect a continuous evolution in oral drug delivery approaches rather than a disruptive transformation, with many strategies already progressing from experimental stages toward industrial application. The integration of advanced materials, tailored release kinetics, and pharmacokinetic optimization underscores the role of MR formulations as an important and expanding component of contemporary pharmacotherapy, particularly for chronic pain and inflammatory conditions.

## Figures and Tables

**Table 1:** Overview of the selected publications included in the review, presenting modified-release oral formulations of non-steroidal anti-inflammatory drugs (NSAIDs), along with the intended effect and formulation technique.

API	Dosage Form Type	Polymer(s)/Material(s)	Release Kinetics/Mechanism	Main Outcomes	Ref.
*Aceclofenac*	Press-coated IR core (pulsatile)	Core: aceclofenac + croscarmellose; Coat: HPMC K100M + HPMC E5 (12.5:87.5)	Pulsatile: lag = 5 h → burst (~99%/6 h)	Core disintegration 15 s; assay 99.9%; hardness 5.5 kp; optimized coat E6 stable ≥ 3 months (ICH); chrono-adapted to morning RA pain	[23]
*Aceclofenac*	Matrix pellets (extrusion–spheronization)	Eudragit RL100; PVP K90	pH-dependent sustained	≤10% (pH 1.2, 2 h); prolonged at pH 6.8–7.4; ↓ gastric irritation vs. control	[24]
*Acetylsalicylic acid*	Enteric-coated pellets (ES + fluidized-bed coat)	ASA/MCC/L-HPC/PVP; enteric: Eudragit L30D-55/TEC; L-Glu companion pellets	pH-triggered (intestinal)	ASA 1.8% (2 h, pH 1.0) → 88.8% (45 min, pH 6.8); L-Glu 98.9% (45 min, pH 7.2); sphericity 0.93–0.94; free SA 0.24%; GI protection confirmed (rat)	[25]
*Acetylsalicylic acid*	Nanoparticles (solvent evaporation)	Sodium alginate; PVP; DCM	Diffusion + anomalous transport	Size 76–128 nm; PDI ≤ 0.46; ζ +36 to +48 mV; EE 36–44%; release 25–36%/2 h → 97%/24 h; antioxidant activity; significant in vivo antithrombotic effect (dose-dependent)	[26]
*Celecoxib*	Mucoadhesive microspheres (ionotropic gelation)	Sodium alginate; CM-chitosan; CaCl_2_	Higuchi (R^2^ = 0.996); KP (*n* = 0.45–0.65, anomalous); sustained 24 h	Size 153–230 μm; EE 59–84%; DL 9–14%; release = 87%/24 h (no burst); mucoadhesion 59%/8 h; amorphous drug	[27]
*Celecoxib*	Functionalized MSNs	APTES-MSN + imidazolyl-PEI gatekeepers	pH-dependent (higher at pH 5.5)	Size ~216 nm; PDI ~0.35; ζ +20 mV; LC 13.5%, EE 13.2%; 2 h: 21% (pH 7.4) vs. 33% (pH 5.5); ↓ PEI cytotoxicity; strongest ↓NO in RAW 264.7	[28]
*Celecoxib*	Hydrogel beads (ionotropic gelation)	Chitosan; guar gum; crosslinked TPP ± glutaraldehyde	KP (*n* = 0.50–0.62, non-Fickian); sustained ≤24 h	Size 1.2–2.7 mm; EE 44–55%; SC: 74%/24 h (pH 7.5) & 24% (pH 1.2); DC: ≤24%/24 h; DC > SC mucoadhesion; Papp ↑; in vivo ↓ CRP/IL-6	[29]
*Celecoxib*	Nano-lyophilized orally disintegrating tablets (ODTs)	PVP K30 (11.3%); SDS (2.6%); mannitol (36.6%)	Rapid dissolution (>90% in 3 min)	Tablet disintegration ≤ 5 s; nanosized CXB (~ 351 nm); solubility ↑ at all pH; relative BA: 155% (rat), 292% (dog) vs. Celebrex^®^; Tmax ↓ 25–33%	[30]
*Diclofenac sodium*	Alginate–LDH microbeads	Sodium alginate; Mg–Al LDH (intercalated DCF); CaCl_2_	Baker–Lonsdale; pH/temperature-responsive SR	Size ~0.9 mm; release 29%/24 h (pH 2.5) → 64% (pH 12); 41%/24 h (25 °C) → 64% (45 °C); plateau ~96 h; Ea 43.5 kJ/mol	[31]
*Diclofenac sodium*	IPN hydrogel microspheres	Pectin; taro polysaccharide; CaCl_2_	Higuchi (R^2^ = 0.99); KP (*n* = 0.48–0.61); SR (T90% ≥ 11 h)	Size 100–130 μm; EE ≤ 91.8%; 60–80%/12 h; no burst	[9]
*Diclofenac sodium*	Matrix tablets (direct compression)	Kollidon^®^ SR; MCC; PVA100; lactose	KP (*n* = 0.45–0.60, non-Fickian); SR ≤ 24 h	100 mg/tablet; Ø13 mm; quaternary KSR–PVA100–MCC matched reference SR (f_2_ > 50); strong/low-porosity; once-daily feasible	[32]
*Diclofenac sodium*	HME matrix	Ethylcellulose; Natrosol L; PEG 8000	Controlled, tunable by polymer/API	Up to 92%/16 h; profile matches reference; tunable via ratios	[18]
*Diclofenac sodium*	Silk fibroin nanoparticles (PVP K30 functionalized)	Silk fibroin; PVP K30	<20% (pH 1.2, 2 h); biphasic at pH 6.8	Size 400–800 nm; ζ −17 to −19 mV; EE ~40% (solvent exchange); PVP 23–50%; in vivo anti-inflammatory + 20–30%; faster onset (1 h)	[33]
*Diclofenac sodium*	Modified polysaccharide nanoparticles	Cashew-gum polysaccharide (CGP)-g-PPG	KP (R^2^ = 0.998; *n* = 0.84); SR ≤ 68 h	Size 275–321 nm; PDI 0.34; ζ ~−6 mV; EE 95.6%; 41%/50 h → plateau 68 h; no burst	[34]
*Diclofenac sodium*	Bilayer IR/SR tablet (HME + FDM 3DP)	IR: PVA (50% DCF, honeycomb); SR: PVA ± Kollidon^®^ SR (14–24%)	KP (*n* ≤ 0.45, Fickian); biphasic	IR 62–68%/30 min; SR ≤ 90%/24 h (Kollidon 19–24%); customizable; Ph. Eur. mass uniformity	[35]
*Etoricoxib (+ famotidine)*	Floating monolayer (gas-generating)	Konjac/guar/xanthan; HPMC K15M; NaHCO_3_	KP (*n* = 0.698); gastric retention ≥ 8 h	Swelling 227–357%/8 h; ET 22%/1 h, 77%/8 h; FM 25%/1 h, 94%/8 h; friability <1 %; AUC_0_–_72_ ↑ = 2× vs. IR	[15]
*Flurbiprofen*	IPN beads (ionic + GA)	Sodium alginate; PVA; methylcellulose; GA	Higuchi (R^2^ 0.95–0.99); KP (*n* = 0.50–0.67)	Size 713–1737 μm; EE 12.7–18.0%; ≤10%/2 h (pH 1.2); up to ~100%/6 h (pH 7.4); more crosslinking → slower	[36]
*Flurbiprofen*	Temperature-responsive nanospheres	Chitosan; HPC; GA; Span 80	KP (*n* = 0.71–1.15); LCST = 42 °C	Size 894–1140 nm; ζ +23–70 mV; EE 22–43%; 24 h: 37–99% (formulation-dependent); 30 °C: 83–88% vs. 44 °C: 52–60%	[37]
*Flurbiprofen*	Polymeric NPs + graphene oxide	Chitosan; graphene oxide; GA; Span 80	Biphasic: burst (46–80%/7 h) → SR (53–74%/24 h); KP (*n* = 0.64–1.26)	Size 362–718 nm; ζ −7.7 to −27.9 mV; EE 19–38%; ↑ GA → slower; ↑ Span 80 → faster; thermo-responsive	[38]
*Ibuprofen*	Solid-lipid microparticles (microfluidic)	Beeswax; Suppocire NAI 25A	Higuchi (R^2^ > 0.99); KP (*n* = 0.44–0.53)	Size ~1 mm; PDI ~0.1; EE ≤ 101%; 50–65%/72 h; SR ≤ 10 days; eutectic in SPC	[22]
*Ibuprofen*	Semi-IPN beads (Fe^3+^ crosslinked)	Sodium alginate; methylcellulose; FeCl_3_	Higuchi; KP (*n* = 0.36–1.09)	Size 1.2–2.0 mm; EE ≤ 93%; ≤15%/2 h (pH 1.2); 80–94%/6 h (pH 7.4); composition/crosslink-time controlled	[39]
*Ibuprofen*	Polyelectrolyte complexes	Chitosan; xanthan gum	Zero-order achievable; KP (*n* = 0.45–0.89)	Yield 48–64%; EE ≤ 62%; ≤10%/2 h (pH 1.2); 60–70%/12 h (pH 7.2)	[40]
*Ibuprofen*	IPN beads	CM-konjac glucomannan; Na-CMC; AlCl_3_	Zero-order (R^2^ 0.97–0.99); KP (*n* = 0.86–0.99)	Size 324–580 μm; yield 85–95%; EE 75–95%; ≤10%/2 h (pH 1.2); ≥ 80%/24 h (pH 7.4)	[10]
*Ibuprofen (+ [Lid][Ibu])*	Thermoplastic zein matrix	Zein; [Lid][Ibu] API-IL ± glycerol	KP (0.5 < *n* < 1); swelling/diffusion-controlled; pH-selective	Swelling 3–4×; Eʹ drop 12→2 MPa; [Lid]^+^: 35%/2 h (SGF), 60–70%/4 h; [Ibu]^−^: ~5%/2 h (SGF), 50–70%/2 h (SIF)	[41]
*Ibuprofen + rabeprazole*	Core–shell (enteric core + IR shell)	IBU core: Eudragit L30D-55; RAB shell	Sequential: IR RAB + delayed pH-triggered IBU	RAB 99.5%/1 h (pH 1.2); IBU 3.4%/2 h (pH 1.2) → 88%/45 min (pH 6.8); dogs: IBU AUC ↑~ 1.7–1.9×; stable ≥ 24 mo	[5]
*Ibuprofen*	OMMT-reinforced alginate beads	Alginate; Ca^2+^; OMMT (CTAB/NPE)	Sustained; KP (*n* = 0.77–0.83)	LC ≤ 5.9%; EE 94.4%; adsorption 28.2 mg/g; ~70–85%/72 h; reduced burst vs. alginate	[42]
*Ibuprofen*	Alginate aerogel beads (Ca^2+^/Ba^2+^)	Sodium alginate; Ca^2+^/Ba^2+^	pH-responsive; 1st-order; KP (*n* = 0.59–0.96)	EE ≤ 95%; porosity 58–79%; <20%/48 h (pH 1.2); 96.9%/1 h (pH 7.2); Ba^2+^ stabilizes in acid	[43]
*Ibuprofen*	3D-printed tablets (LCD, visible light)	PEGDA; PEG 400; water; riboflavin	Sustained; KP (*n* < 0.45, diffusion)	Amorphous IBU; 100%/6–7 h (450 nm) vs. 40–85% (405 nm); drug load 5–6% (22% with high-water resin)	[11]
*Ibuprofen*	Liposomes (CD-assisted remote loading)	DSPC:Chol:PEG-DSPE; intraliposomal SBE-*β*-CD	Diffusion-controlled; burst suppression by CD	Size 82–146 nm; PDI < 0.25; EE: 7% (hydration) → 27% (pH-gradient) → 80% (CD 200 mM); burst 62%/4 h without CD	[44]
*Ibuprofen*	Direct-compressed matrix	Irvingia gabonensis (vs. HPMC/xanthan)	Super case-II (*n* > 1.0); SR	t25 1.05–3.6 h (IG) faster than HPMC/xanthan; CSFR 2.6–24; natural, low-cost CR polymer	[45]
*Ibuprofen; Diclofenac sodium*	Core–shell magnetic LDH NPs	Fe_3_O_4_ core; Mg/Al-LDH shell	Sustained ≤ 72 h; surface diffusion + anion exchange	IBU 90%/24 h, 96%/72 h; DCF 78%/24 h, 82%/72 h; basal spacing 2.62 nm (IBU), 2.22 nm (DCF)	[46]
*Ibuprofen*	Floating asymmetric-membrane NPs	Ethylcellulose; HPMC E15LV; glycerol; Tween 20	Gastro-retentive; Higuchi (R^2^ = 0.99); Fickian (*n* = 0.05)	Size 114–167 nm; loading 97.4%; buoyancy > 12 h (no lag); solubility ~2× vs. raw; anti-inflammatory 85% vs. 78%	[47]
*Ibuprofen*	Radially porous silica NPs (agarose-coated)	Mesoporous silica + APTES; agarose coating	pH-dependent; temp-responsive; SR ≤ 300 h	Loading 270 wt% (2.7 *g*/*g*); ~40%/50 h (pH 2) vs. ~80%/50 h (pH 12); non-toxic to fibroblasts	[48]
*Ibuprofen*	Prolonged-release capsules	—(commercial SR excipients)	Sustained; plateau PK	Fasting: Cmax 14.9 μg/mL @ 5 h, AUC_0_–t 105; Fed: Cmax 21.3 μg/mL @ 5.6 h, AUC_0_–t 113; bioequivalent (90% CI in 80–125%); safe	[49]
*Ibuprofen*	Silica aerogel (surface-modified)	SA; SA@APTES (hydrophilic) or SA@TMCS (hydrophobic)	Controlled; surface-chemistry modulated	Amorphization 85–100%; stability ≥ 6 m; SA/APTES: 80% in 1–10 min; TMCS: 80% in 3–24 h	[50]
*Ibuprofen*	Alginate hydrogel + Fe-cellulose nanobiocomposite	Alginate; Fe-CNB ± *β*-CD	pH-dependent via charge reversal	DLE: CA 23%, *β*-CD 46%, Fe-CNB 41%, Fe-CNB+*β*-CD 47%; 20% (pH 1.2, 2 h) vs. 49% (pH 7.4, 12 h)	[51]
*Ibuprofen*	HME amorphous solid dispersion	RSPO + PVP VA64 (35–50%); comps. with EC/Soluplus	Fickian diffusion (*n* = 0.10–0.26); dissolution plateau	~43%/1 h; 70%/12 h; 88%/24 h stable across 35–50% VA64; plateau reduces batch variability	[19]
*Ibuprofen*	Thin films (spin-coating vs. HME)	Cellaburate/rosin (65:35) + 30% IBU	First-order; KP 0.5–1.2; Weibull (b > 1)	Amorphous; spin: >90%/1 h (burst); HME: ~100%/=5 h; film thickness 82 μm (spin) vs. 1.6 mm (HME); stability ≥ 3 m (spin)	[52]
*Ibuprofen*	Ionotropically gelled microspheres	Na-alginate + plant mucilage/pectin; ZnCl_2_	Ratio-dependent	SR at polymer ratio 1:1; IR at 1:2; EE ≤ 60.4%; no drug–polymer interaction	[53]
*Ibuprofen*	Dual-nozzle electrospun nanofibers	PVP (fast) + HPMC (slow)	Biphasic: ~40% @ 5 min + SR ≤ 12 h	Fiber 316 ± 29 nm; amorphous; stable 1 m @ 40 °C; in vivo ulcers: 1.8 ± 0.5 vs. 8.8 ± 1.5 (*p* < 0.01)	[20]
*Ibuprofen*	Matrix tablets with ASD-IBP	ASD (IBP + Kolliphor P407); matrix: Kollidon SR/Eudragit RSPO	Modified SR ~12 h; Weibull best; KP n = 0.52–0.74	Solubility ↑ 28–35×; 69–88%/12 h; MDT 3.6–5.6 h; hardness 3.5–6.2 kg/cm^2^; friability < 0.5%	[54]
*Indomethacin*	Core–shell composite fibers (wet-spun)	Sodium alginate; feather keratin	Sustained	≤80%/12 h (intestinal media); keratin fraction modulates release	[55]
*Indomethacin*	RAFT nanomicelles	Dextran-g-PMABTE	pH-dependent; prolonged	88%/48 h; faster at pH 7.4; LC 24.1%; EE 96%	[56]
*Indomethacin*	Self-assembled microspheres	κ-Carrageenan; sericin (ionotropic)	pH-responsive; mucoadhesive	~90%/24 h (formulation-dependent); high loading ≤ 54%; strong mucoadhesion; biocompatible	[16]
*Indomethacin*	Buccal matrix tablets	Chitosan (10–20%); Carbopol (5–15%); HPMC; fillers	Diffusion + swelling; KP *n* = 0.65–1.31	Hardness ↑ with PAA (~100 N); swelling up to 7.67 (10% PAA)/8 h; mucoadhesion ≤ 0.79 N; 27–39%/12 h (10% PAA)	[17]
*Indomethacin*	Pellets → ODT matrix	Eudragit L100 pellets in ODT	pH-dependent	Inhibited at pH 1.2; intense at pH 6.8; pellets intact after compression; ODT disintegration < 30 s	[57]
*Indomethacin*	PLGA microparticles (microfluidic + ANN optimization)	PLGA	Biphasic; sustained (80%/9 d)	Size-tunable monodisperse MPs; EE ~62%, DL ~7.8%; ANN accurately predicted size; reproducible long-term release	[58]
*Ketoprofen*	Enteric-coated (100 mg) & XR (200 mg)	EC: PVAP or MMA; XR: HPMC/HEC/MCC ± DCP	EC: pH-triggered; XR: dissolution-controlled, buffer-dependent	EC acid resistance; MDT 24–46 min (buffer-dependent); XR: ~60%/3 h, 90%/6 h (USP); slower in citrate/succinate; DCP raises microclimate pH & solubility 1.5–2.3×; XR BA ~92%	[14]
*Ketoprofen*	3D-printed multilayer tablets (FDM)	PVA filament with KTP (HME); ± Kollicoat IR layer	Profile-switchable depending on infill/layer	Filament amorphous; mechanical strength 466–2141 N/mm^2^; T3: 84%/3 h; T11: 62%/3 h; 20% infill → 2.3–3.3× faster; with Kollicoat IR: 70–80%/2 h	[59]
*Ketoprofen*	Matrix tablets (surfactant-assisted WG)	Soluplus (3%)	KP *n* = 0.45–0.56; SR ≥ 24 h	2.29-fold ↑ bioavailability vs. control; once-daily feasible	[60]
*Ketoprofen*	Hot-extruded pellets	Eudragit L100; Eudragit L100-55; Stearic acid	pH-dependent	<5% in SGF (120 min); >85–95% in SIF (30–45 min); stable pellets	[61]
*Ketorolac*	pH-responsive hydrogel	Chondroitin sulfate; Pluronic F-127; acrylic acid; APS; MBA	Zero-order; pH-dependent	Minimal release in acid; gradual up to 36 h at pH 7.4; high swelling/porosity; crosslinked network	[62]
*Ketorolac*	Compression-coated pulsatile tablet	PEO WSR; Eudragit RLPO	Pulsatile (lag = 9 h → release)	95% released within 17.4 h; ↑ bioavailability vs. solution; controlled lag	[12]
*Lornoxicam*	Biphasic compression-coated tablet	IR: PVP K30 nanocrystals; SR: HPMC matrix	IR + zero-order SR	Nanocrystal size 279 nm; solubility ↑ 3×; IR: disintegration 30 s, 58%/5 min; SR: zero-order (20%/2 h, 80%/8 h, f_2_ = 86); dog PK: Cmax 5.1 vs. 3.7 µg/mL, relative BA 109%	[63]
*Mefenamic acid*	Multiparticulate gel beads	κ-Carrageenan; sericin; PA/DSP/CMC	Prolonged ≤ 48 h (with CMC)	<10%/2 h (pH 1.2); prolonged release at pH 7.4; polymer compatibility confirmed; stable structure	[64]
*Meloxicam*	Multimolecular granules (multi-stage WG)	Lactose; Eudragit NM/FS; Metolose	Delayed (pH ≥ 6.8); no initial burst	Release ≤ 98.5%/8 h; acid protection; stable granules	[65]
*Meloxicam*	Electrospun nanofibers → SR tablets	HPMC-AS; HPMC K100LV	Tunable; acid-resistant after coating	Fiber size ~0.39 μm; solubility ↑ across pH 1–7.2; coated tablets prevented release at pH 1.0 (2 h) and 4.5 (4 h); gradual at pH 7.2; ↓ gastric irritation risk	[21]
*Naproxen*	Microspheres (CaCl_2_ gelation)	Sericin; alginate; ± PVA/PEG/DSP	Delayed (acid) + SR (≤6 h intestinal)	EE 84–89%; DL 18–30%; <10%/2 h (pH 1.2); ~98%/5–6 h (pH 7.4); reduced crystallinity; thermal stability	[66]
*Naproxen*	Hybrid polymeric microspheres → tablets	Eudragit L100; HPMC; SLS	pH-selective sustained	Particle size 29–74 μm; EE 73–92%; ≤5%/2 h (pH 1.2); >85%/6 h (pH 6.8); in vivo: Cmax 44 µg/mL, Tmax 4.3 h, BA ↑ 5.5-fold	[67]
*Paracetamol*	3D-printed tablets (FDM)	PCL or PEO-based filaments (HME)	Polymer-dependent profile	PCL: ≤50%/8 h; PEO 100K/200K: ~100%/4 h; tunable by polymer type	[8]
*Paracetamol*	3D screen-printed IR/ER tablets	IR: PVP/PEG400; ER: Eudragit RL; silica mod.	Programmable IR/ER	IR ≥ 80%/15 min; ER ≥ 80%/95 min; size/weight within Ph. Eur.; reproducible	[7]
*Paracetamol*	Gas microvesicle encapsulation	Hydrophobic silica particles	Bile-triggered release	<0.2 μg/mL at pH 7; complete release with bile (pH 4–7); stable in saliva/gastric fluids; good palatability	[68]
*Paracetamol*	Direct-compressed mesoporous silica tablets	KCC-1 and KCC-1-NH_2_	Surface-modulated sustained	Surface area: 356 vs. 248 m^2^/g; slower release with NH_2_ modification; near-complete within 240 min; diffusion-controlled	[6]
*Zaltoprofen*	Nanostructured lipid carriers (hot-melt homogenization)	GMS (solid lipid); Capryol 90 (liquid lipid); Myrj 52 (surfactant)	Sustained; biphasic	Particle size 105.5 nm; EE 99.8%; ~40%/2 h, ~75%/24 h; Papp ↑ 1.6-fold; oral BA ↑ 4.3-fold (431%)	[69]

Abbreviations: ↓ = decrease/reduction; ↑ = increase/enhancement; ANN = Artificial Neural Network; API = Active Pharmaceutical Ingredient; AUC = Area Under the Curve; BA = Bioavailability; Cmax = Maximum plasma concentration; CRP = C-Reactive Protein; DL = Drug Loading; Ea = Activation Energy; EE = Encapsulation Efficiency; f_2_ = Similarity Factor; FDM = Fused Deposition Modeling; HME = Hot-Melt Extrusion; IL-6 = Interleukin-6; IPN = Interpenetrating Polymer Network; IR = Immediate Release; LC = Loading Capacity; LCST = Lower Critical Solution Temperature; LDH = Layered Double Hydroxide; LCD = Liquid Crystal Display; MDT = Mean Dissolution Time; MSN = Mesoporous Silica Nanoparticles; NO = Nitric Oxide; ODT = Orally Disintegrating Tablet; Papp = Apparent Permeability; PDI = Polydispersity Index; PK = Pharmacokinetics; RAFT = Reversible Addition–Fragmentation Chain Transfer; SR = Sustained Release; Tmax = Time to maximum concentration; XR = Extended Release; ζ = zeta potential.

## Data Availability

No new data were created or analyzed in this study.

## Supplementary Materials

The following supporting information can be downloaded at: https://www.mdpi.com/article/10.3390/pharmaceutics17101264/s1, PRISMA 2020 Main Checklist. Ref. [73] is cited within.

## References

[1] Majumder J., Taratula O., Minko T. (2019). Nanocarrier-Based Systems for Targeted and Site Specific Therapeutic Delivery. Adv. Drug Deliv. Rev..

[2] Adepu S., Ramakrishna S. (2021). Controlled Drug Delivery Systems: Current Status and Future Directions. Molecules.

[3] Ahmed N., Ly H., Pan A., Chiang B., Raines K., Janwatin T., Hamed S., Dave K. (2023). Retrospective Analysis of the Biopharmaceutics Characteristics of Solid Oral Modified-Release Drug Products in Approved US FDA NDAs Designated as Extended-Release or Delayed-Release Formulations. Eur. J. Pharm. Biopharm..

[4] Murugesan S., Gowramma B., Lakshmanan K., Reddy Karri V.V.S., Radhakrishnan A. (2019). Oral Modified Drug Release Solid Dosage Form with Special Reference to Design; An Overview. Curr. Drug Res. Rev..

[5] Khan B., Choi H.I., Ryu J.S., Noh H.Y., Shah F.A., Khan N., Ansari M.M., Zeb A., Kim J.K. (2024). Core-Shell Tablets Designed for Modified and Sequential Release of Ibuprofen and Rabeprazole. Int. J. Pharm..

[6] Pishnamazi M., Hafizi H., Pishnamazi M., Marjani A., Shirazian S., Walker G.M. (2021). Controlled Release Evaluation of Paracetamol Loaded Amine Functionalized Mesoporous Silica KCC1 Compared to Microcrystalline Cellulose Based Tablets. Sci. Rep..

[7] Enke M., Schwarz N., Gruschwitz F., Winkler D., Hanf F., Jescheck L., Seyferth S., Fischer D., Schneeberger A. (2023). 3D Screen Printing Technology Enables Fabrication of Oral Drug Dosage Forms with Freely Tailorable Release Profiles. Int. J. Pharm..

[8] Đuranović M., Obeid S., Madžarević M., Cvijić S., Ibrić S. (2021). Paracetamol Extended Release FDM 3D Printlets: Evaluation of Formulation Variables on Printability and Drug Release. Int. J. Pharm..

[9] Sarkar S., Manna S., Das E., Jana P., Mukherjee S., Sahu R., Dua T.K., Paul P., Kaity S., Nandi G. (2024). Fabrication and Optimization of Extended-Release Beads of Diclofenac Sodium Based on Ca++ Cross-Linked Taro (*Colocasia esculenta*) Stolon Polysaccharide and Pectin by Quality-by-Design Approach. Int. J. Biol. Macromol..

[10] Lohani A., Saxena R., Khan S., Mascarenhas-Melo F. (2024). PH-Responsive IPN Beads of Carboxymethyl Konjac Glucomannan and Sodium Carboxymethyl Cellulose as a Controlled Release Carrier for Ibuprofen. Int. J. Biol. Macromol..

[11] Madžarević M., Ibrić S. (2021). Evaluation of Exposure Time and Visible Light Irradiation in LCD 3D Printing of Ibuprofen Extended Release Tablets. Eur. J. Pharm. Sci..

[12] Aldawsari H.M., Naveen N.R., Alhakamy N.A., Goudanavar P.S., Rao G.K., Budha R.R., Nair A.B., Badr-Eldin S.M. (2022). Compression-Coated Pulsatile Chronomodulated Therapeutic System: QbD Assisted Optimization. Drug Deliv..

[13] Cong D., Qi W., Liu X., Xu X., Dong L., Xue W., Li K. (2023). Pharmacokinetic Study of Enteric-Coated Sustained-Release Aspirin Tablets in Healthy Chinese Participants. Drug Des. Devel Ther..

[14] García M.A., Al-Gousous J., González P.M., Langguth P. (2025). Model-Supported Dissolution Methods for Modified-Release Products: Enteric-Coated versus Extended-Release Ketoprofen Tablets. Int. J. Pharm..

[15] Saady M., Shoman N.A., Teaima M., Abdelmonem R., El-Nabarawi M.A., Elhabal S.F. (2024). Fabrication of Gastro-Floating Sustained-Release Etoricoxib and Famotidine Tablets: Design, Optimization, in-Vitro, and in-Vivo Evaluation. Pharm. Dev. Technol..

[16] Vieira W.T., Viegas J.S.R., da Silva M.G.C., de Oliveira Nascimento L., Vieira M.G.A., Sarmento B. (2024). Self-Assembly Mucoadhesive Beads of κ-Carrageenan/Sericin for Indomethacin Oral Extended Release. Int. J. Biol. Macromol..

[17] Esim O., Savaser A., Ozkan C.K., Tas C., Ozkan Y. (2020). Investigation of the Mucoadhesivity, Swelling, and Drug Release Mechanisms of Indomethacin Buccal Tablets: Effect of Formulation Variables. Drug Dev. Ind. Pharm..

[18] Sanoufi M.R., Aljaberi A., Hamdan I., Al-Zoubi N. (2020). The Use of Design of Experiments to Develop Hot Melt Extrudates for Extended Release of Diclofenac Sodium. Pharm. Dev. Technol..

[19] Chen L., Hu E., Shen P., Qian S., Heng W., Zhang J., Gao Y., Wei Y. (2024). Development of Amorphous Solid Dispersion Sustained-Release Formulations with Polymer Composite Matrix-Regulated Stable Release Plateaus. Pharm. Res..

[20] Che X., Xue J., Zhang J., Yang X., Wang L. (2020). One-Step Preparation of Ibuprofen Fast- and Sustained-Release Formulation by Electrospinning with Improved Efficacy and Reduced Side Effect. Pharm. Dev. Technol..

[21] Friuli V., Pisani S., Conti B., Bruni G., Maggi L. (2022). Tablet Formulations of Polymeric Electrospun Fibers for the Controlled Release of Drugs with PH-Dependent Solubility. Polymers.

[22] Ho L.Y., Xiang Z.S., Gopal R., Khan S.A. (2021). Microfluidics-Enabled Particle Engineering of Monodisperse Solid Lipid Microparticles with Uniform Drug Loading and Diverse Solid-State Outcomes. Int. J. Pharm..

[23] Rashid R., Zaman M., Ahmad M., Khan M.A., Butt M.H., Salawi A., Almoshari Y., Alshamrani M., Sarfraz R.M. (2022). Press-Coated Aceclofenac Tablets for Pulsatile Drug Delivery: Formulation and In Vitro Evaluations. Pharmaceuticals.

[24] Ibrahim M.A., Alshora D.H. (2021). Development and Characterization of Eudragit-Rl-100-Based Aceclofenac Sustained-Release Matrix Pellets Prepared via Extrusion/Spheronization. Polymers.

[25] Xu M., Liu F., Zhou W., He B., Tan S. (2021). Preparation and Preliminary Quality Evaluation of Aspirin/L-Glutamate Compound Pellets. J. Mater. Sci. Mater. Med..

[26] Alhajj L., Airemwen C.O., Pozharani L.B. (2023). Formulation of Aspirin Nanoparticles Using Solvent Evaporation Method and in Vivo Evaluation of Its Antithrombotic Effect. Pak. J. Pharm. Sci..

[27] Biji C.A., Balde A., Kim S.K., Nazeer R.A. (2024). Optimization of Alginate/Carboxymethyl Chitosan Microbeads for the Sustained Release of Celecoxib and Attenuation of Intestinal Inflammation in Vitro. Int. J. Biol. Macromol..

[28] Mudhakir D., Sadaqa E., Permana Z., Mumtazah J.E., Zefrina N.F., Xeliem J.N., Hanum L.F., Kurniati N.F. (2024). Dual-Functionalized Mesoporous Silica Nanoparticles for Celecoxib Delivery: Amine Grafting and Imidazolyl PEI Gatekeepers for Enhanced Loading and Controlled Release with Reduced Toxicity. Molecules.

[29] Batool R., Mudassir J., Khan M.A., Zafar S., Rana S.J., Abbas N., Hussain A., Arshad M.S., Muhammad S. (2023). Fabrication and Characterization of Celecoxib-Loaded Chitosan/Guar Gum-Based Hydrogel Beads. Pharmaceuticals.

[30] Sun S., Wang M., Chen J., Ju X., Zhang F., He M., Cheng D., Kong S. (2025). Preparation and Evaluation of Celecoxib Lyophilized Orally Disintegrating Tablets with High Bioavailability. Eur. J. Pharm. Biopharm..

[31] Viscusi G., Gorrasi G. (2021). Facile Preparation of Layered Double Hydroxide (LDH)-Alginate Beads as Sustainable System for the Triggered Release of Diclofenac: Effect of PH and Temperature on Release Rate. Int. J. Biol. Macromol..

[32] Obeidat W.M., Lahlouh I.K., Gharaibeh S.F. (2023). Investigations on Compaction Behavior of Kollidon^®^SR-Based Multi-Component Directly Compressed Tablets for Preparation of Controlled Release Diclofenac Sodium. AAPS PharmSciTech.

[33] Di K.N., Ha P.T.M., Nguyen N.P., Nguyen N.Y., Truong T.C., Nguyen T.T.V., Truong Q.K., Nguyen M.Q., Pham D.T. (2025). Enhanced Anti-Inflammatory Effects of Diclofenac Delivered Orally via Polyvinylpyrrolidone K30/Silk Fibroin Nanoparticles in a Murine Model of Carrageenan-Induced Paw Edema. ChemMedChem.

[34] da Silva C.N.S., Di-Medeiros M.C.B., Lião L.M., Fernandes K.F., Batista K.d.A. (2021). Cashew Gum Polysaccharide Nanoparticles Grafted with Polypropylene Glycol as Carriers for Diclofenac Sodium. Materials.

[35] Crișan A.G., Porfire A., Iurian S., Rus L.M., Lucăcel Ciceo R., Turza A., Tomuță I. (2023). Development of a Bilayer Tablet by Fused Deposition Modeling as a Sustained-Release Drug Delivery System. Pharmaceuticals.

[36] Bulut E. (2020). Flurbiprofen-Loaded Interpenetrating Polymer Network Beads Based on Alginate, Polyvinyl Alcohol and Methylcellulose: Design, Characterization and in-Vitro Evaluation. J. Biomater. Sci. Polym. Ed..

[37] Işıklan N., Erol Ü.H. (2020). Design and Evaluation of Temperature-Responsive Chitosan/Hydroxypropyl Cellulose Blend Nanospheres for Sustainable Flurbiprofen Release. Int. J. Biol. Macromol..

[38] Erol Ü.H., Güncüm E., Işıklan N. (2023). Development of Chitosan-Graphene Oxide Blend Nanoparticles for Controlled Flurbiprofen Delivery. Int. J. Biol. Macromol..

[39] Bulut E. (2021). Development and Optimization of Fe^3+^-Crosslinked Sodium Alginate-Methylcellulose Semi-Interpenetrating Polymer Network Beads for Controlled Release of Ibuprofen. Int. J. Biol. Macromol..

[40] Ćirić A., Medarević Đ., Čalija B., Dobričić V., Rmandić M., Barudžija T., Malenović A., Djekic L. (2021). Effect of Ibuprofen Entrapment Procedure on Physicochemical and Controlled Drug Release Performances of Chitosan/Xanthan Gum Polyelectrolyte Complexes. Int. J. Biol. Macromol..

[41] Thadasack M., Chaunier L., Rabesona H., Viau L., De-Carvalho M., Bouchaud G., Lourdin D. (2022). Release Kinetics of [Lidocainium][Ibuprofenate] as Active Pharmaceutical Ingredient-Ionic Liquid from a Plasticized Zein Matrix in Simulated Digestion. Int. J. Pharm..

[42] Yan H., Chen X., Bao C., Yi J., Lei M., Ke C., Zhang W., Lin Q. (2020). Synthesis and Assessment of CTAB and NPE Modified Organo-Montmorillonite for the Fabrication of Organo-Montmorillonite/Alginate Based Hydrophobic Pharmaceutical Controlled-Release Formulation. Colloids Surf. B Biointerfaces.

[43] Li Y., Fan R., Xing H., Fei Y., Cheng J., Lu L. (2021). Study on Swelling and Drug Releasing Behaviors of Ibuprofen-Loaded Bimetallic Alginate Aerogel Beads with PH-Responsive Performance. Colloids Surf. B Biointerfaces.

[44] Akaki S., Hosokawa M., Maeda S., Kono Y., Maeda H., Ogawara K.I. (2024). Efficient Loading into and Controlled Release of Lipophilic Compound from Liposomes by Using Cyclodextrin as Novel Trapping Agent. Biol. Pharm. Bull..

[45] Patani B.O., Akin-Ajani O.D., Kumaran A., Odeku O.A. (2022). Irvingia Gabonensis (O’Rorke) Bail Polymer Matrix System for Controlled Drug Delivery. Polim. Med..

[46] Yousefi V., Tarhriz V., Eyvazi S., Dilmaghani A. (2020). Synthesis and Application of Magnetic@layered Double Hydroxide as an Anti-Inflammatory Drugs Nanocarrier. J. Nanobiotechnol..

[47] Samuel B.A., Mohammed B.I., Philip A.K. (2021). Phase Transited Asymmetric Membrane Floating Nanoparticles: A Means for Better Management of Poorly Water-Soluble Drugs. DARU J. Pharm. Sci..

[48] Choi Y., Kim J., Yu S., Hong S. (2020). PH-and Temperature-Responsive Radially Porous Silica Nanoparticles with High-Capacity Drug Loading for Controlled Drug Delivery. Nanotechnology.

[49] Huang C., Yin Z., Yang Y., Mo N., Yang H., Wang Y. (2023). Evaluation of Pharmacokinetics and Safety with Bioequivalence of Ibuprofen Sustained-Release Capsules of Two Formulations, in Chinese Healthy Volunteers: Bioequivalence Study. Drug Des. Devel Ther..

[50] Zarinwall A., Maurer V., Pierick J., Oldhues V.M., Porsiel J.C., Finke J.H., Garnweitner G. (2022). Amorphization and Modified Release of Ibuprofen by Post-Synthetic and Solvent-Free Loading into Tailored Silica Aerogels. Drug Deliv..

[51] Varghese S., Chaudhary J.P., Thareja P., Ghoroi C. (2023). Newly Developed Nano-Biocomposite Embedded Hydrogel to Enhance Drug Loading and Modulated Release of Anti-Inflammatory Drug. Pharm. Dev. Technol..

[52] Albarahmieh E., Alkhalidi B.A., Al-Hiari Y. (2020). Evaluation of Amorphous Dispersion of a Cellulose Ester-Colophony Mix for Ibuprofen Controlled Release Processed by HME and Spin Coating. Carbohydr. Polym..

[53] Akin-Ajani O.D., Hassan T.M., Odeku O.A. (2022). *Talinum triangulare* (Jacq.) Willd. Mucilage and Pectin in the Formulation of Ibuprofen Microspheres. Polim. Med..

[54] Uddin A., Halder S., Deb N., Das H., Shuma M.L., Hasan I., Shill M.C., Haider S.S. (2024). Impact of Methods of Preparation on Mechanical Properties, Dissolution Behavior, and Tableting Characteristics of Ibuprofen-Loaded Amorphous Solid Dispersions. Adv. Pharmacol. Pharm. Sci..

[55] Gong X., Dang G., Guo J., Liu Y., Gong Y. (2020). A Sodium Alginate/Feather Keratin Composite Fiber with Skin-Core Structure as the Carrier for Sustained Drug Release. Int. J. Biol. Macromol..

[56] Karmakar P.D., Pal S. (2021). Dextran Based Amphiphilic Self-Assembled Biopolymeric Macromolecule Synthesized via RAFT Polymerization as Indomethacin Carrier. Int. J. Biol. Macromol..

[57] Al-hashimi N., Dahmash E.Z., Khoder M., Alany R., Elshaer A. (2025). Engineering PH-Dependent Orally Disintegrating Tablets for Modified Indomethacin Release: A Polymer-Based Approach. AAPS PharmSciTech.

[58] Damiati S.A., Damiati S. (2021). Microfluidic Synthesis of Indomethacin-Loaded PLGA Microparticles Optimized by Machine Learning. Front. Mol. Biosci..

[59] Pyteraf J., Jamróz W., Kurek M., Szafraniec-Szczęsny J., Kramarczyk D., Jurkiewicz K., Knapik-Kowalczuk J., Tarasiuk J., Wroński S., Paluch M. (2021). How to Obtain the Maximum Properties Flexibility of 3D Printed Ketoprofen Tablets Using Only One Drug-Loaded Filament?. Molecules.

[60] Shamim R., Shafique S., Hussain K., Abbas N., Ijaz S., Bukhari N.I. (2024). Surfactant-Assisted Wet Granulation-Based Matrix Tablets without Exceptional Additives: Prolonging Systemic Exposure of Model BCS Class II Ketoprofen. AAPS PharmSciTech.

[61] Vo A.Q., Kutz G., He H., Narala S., Bandari S., Repka M.A. (2020). Continuous Manufacturing of Ketoprofen Delayed Release Pellets Using Melt Extrusion Technology: Application of QbD Design Space, Inline Near Infrared, and Inline Pellet Size Analysis. J. Pharm. Sci..

[62] Naeem S., Barkat K., Shabbir M., Khalid I., Anjum I., Shamshad N., Mehmood Y., Khan D.H., Badshah S.F., Syed M.A. (2022). Fabrication of PH Responsive Hydrogel Blends of Chondroitin Sulfate/Pluronic F-127 for the Controlled Release of Ketorolac: Its Characterization and Acute Oral Toxicity Study. Drug Dev. Ind. Pharm..

[63] Tung N.T., Dong T.H.Y., Tran C.S., Nguyen T.K.T., Chi S.C., Dao D.S., Nguyen D.H. (2022). Integration of Lornoxicam Nanocrystals into Hydroxypropyl Methylcellulose-Based Sustained Release Matrix to Form a Novel Biphasic Release System. Int. J. Biol. Macromol..

[64] Vieira W.T., Nicolini M.V.S., da Silva M.G.C., Nascimento L.d.O., Vieira M.G.A. (2024). κ-Carrageenan/Sericin Polymer Matrix Modified with Different Crosslinking Agents and Thermal Crosslinking: Improved Release Profile of Mefenamic Acid. Int. J. Biol. Macromol..

[65] Navarro-Ruíz E., Álvarez-Álvarez C., Peña M.Á., Torrado-Salmerón C., Dahma Z., de la Torre-Iglesias P.M. (2022). Multiparticulate Systems of Meloxicam for Colonic Administration in Cancer or Autoimmune Diseases. Pharmaceutics.

[66] Freitas E.D., Freitas V.M.S., Rosa P.C.P., da Silva M.G.C., Vieira M.G.A. (2021). Development and Evaluation of Naproxen-Loaded Sericin/Alginate Beads for Delayed and Extended Drug Release Using Different Covalent Crosslinking Agents. Mater. Sci. Eng. C.

[67] Hameed H.A., Khan S., Shahid M., Ullah R., Bari A., Ali S.S., Hussain Z., Sohail M., Khan S.U., Htar T.T. (2020). Engineering of Naproxen Loaded Polymer Hybrid Enteric Microspheres for Modified Release Tablets: Development, Characterization, in Silico Modelling and in Vivo Evaluation. Drug Des. Devel. Ther..

[68] Poortinga A.T., van Nostrum C.F. (2025). Microbubble-Encapsulation of Actives for Controlled Release and Its Application to the Taste-Masking of Acetaminophen. Int. J. Pharm..

[69] Pham T.M.A., Lee D.H., Na Y.G., Jin M., Jung M., Kim H.E., Yoo H., Won J.H., Lee J.Y., Baek J.S. (2022). Enhancement of S(+)-Zaltoprofen Oral Bioavailability Using Nanostructured Lipid Carrier System. Arch. Pharm. Res..

[70] Khan M.A., Otero M., Kazi M., Alqadami A.A., Wabaidur S.M., Siddiqui M.R., Alothman Z.A., Sumbul S. (2019). Unary and Binary Adsorption Studies of Lead and Malachite Green onto a Nanomagnetic Copper Ferrite/Drumstick Pod Biomass Composite. J. Hazard. Mater..

[71] Alothman Z.A., Bahkali A.H., Khiyami M.A., Alfadul S.M., Wabaidur S.M., Alam M., Alfarhan B.Z. (2020). Low Cost Biosorbents from Fungi for Heavy Metals Removal from Wastewater. Sep. Sci. Technol..

[72] Anam A., Abbas G., Shah S., Saadullah M., Shahwar D., Mahmood K., Hanif M., Ahmad N., Basheer E., Obaidullah A.J. (2024). Quantitative Analysis of Loxoprofen Sodium Loaded Microspheres Comprising Pectin and Its Thiolated Conjugates: In-Vivo Evaluation of Their Sustained Release Behavior. Heliyon.

[73] Tricco A.C., Lillie E., Zarin W., O’Brien K.K., Colquhoun H., Levac D., Moher D., Peters M.D., Horsley T., Weeks L. (2018). PRISMA Extension for Scoping Reviews (PRISMAScR): Checklist and Explanation. Ann. Intern. Med..

